# Endogenous bacteria inhabiting the *Ophiocordyceps highlandensis* during fruiting body development

**DOI:** 10.1186/s12866-021-02227-w

**Published:** 2021-06-11

**Authors:** Chengpeng Li, Dexiang Tang, Yuanbing Wang, Qi Fan, Xiaomei Zhang, Xiaolong Cui, Hong Yu

**Affiliations:** 1 Yunnan Herbal Laboratory, School of Ecology and Environmental Science, Yunnan University, Kunming, 650091 China; 2 Yunnan Institute of Microbiology, School of Life Sciences, Yunnan University, Kunming, Yunnan 650091 People’s Republic of China; 3 The Research Center of Cordyceps Development and Utilization of Kunming, Yunnan Herbal Biotech Co. Ltd, Kunming, 650106 China; 4 College of Basic Medicine, Yunnan University of Chinese Medicine, Kunming, 650500 China

**Keywords:** Bioinoculants, *Ophiocordyceps highlandensis*, Microbial assemblages, Fruiting body development, Cultivation

## Abstract

**Background:**

The genus *Ophiocordyceps*, which includes *Ophiocordyceps sinensis*, has been demonstrated to be one of the most valuable medicinal taxa. The low rate of larval infection and slow development that characterize the cultivation of this genus should be urgently addressed. To identify potential bioinoculants that stimulate the growth of *Ophiocordyceps*, *O. highlandensis* was selected as a model system, and a total of 72 samples were collected to systematically compare the microbial communities present during fruiting body development. By applying high-throughput 16S and ITS2 amplicon sequencing technology, the bacterial and fungal communities were identified in *O. highlandensis* and its surrounding soil, and the functional dynamics of the bacteria were explored.

**Results:**

The results indicate that the most abundant bacteria across all the samples from *O. highlandensis* were Proteobacteria, Firmicutes and Bacteroidetes, while members of Ascomycota were detected among the fungi. The pathways enriched in the developmental stages were associated with carbohydrate degradation, nucleotides and pyridoxal biosynthesis, and the TCA cycle. Compared with that in the fungal community, an unexpectedly high taxonomic and functional fluctuation was discovered in the bacterial community during the maturation of *O. highlandensis*. Furthermore, bipartite network analysis identified four potential supercore OTUs associated with *O. highlandensis* growth.

**Conclusions:**

All the findings of this study suggest unexpectedly high taxonomic and functional fluctuations in the bacterial community of *O. highlandensis* during its maturation. *O. highlandensis* may recruit different endogenous bacteria across its life cycle to enhance growth and support rapid infection. These results may facilitate *Ophiocordyceps* cultivation and improve the development of strategies for the identification of potential bioinoculant resources.

**Supplementary Information:**

The online version contains supplementary material available at 10.1186/s12866-021-02227-w.

## Background

The members of the genus *Ophiocordyceps* represent the most valuable and widely acknowledged resources for traditional medicine in China. Currently, over 20 bioactive components have been detected from these fungi [1]. In particular, *O. sinensis* exhibits various pharmacological effects against cancer and inflammatory [2] and cardiovascular diseases [3]; it also has antioxidant effects [4]. However, increasing social demand and the decreasing supply of natural *O. sinensis* may soon cause its extinction. Though considerable progress has been achieved in the cultivation of *O. sinensis*, substantial challenges remain, including low larval infection and primordium induction rates as well as slow development. It is of utmost significance to resolve these issues and increase the yields of these species under cultivation.

Fungi-selected microbiotas act as critical components and significantly impact the growth and development of and bioactive substance production by macromycetes. A recent study reported that aseptic environments do not allow macrofungi to produce fruiting bodies [5]. It has been demonstrated that bacteria enhance fungal mycelial growth and spore germination [6]. Other studies reported that some fungi present in *O. sinensis* may be required for stroma formation [7]. Moreover, the available knowledge suggests that some fungal cultivation strategies rely on the exploitation of specialized host microbes during infection [8]. As a result, experiments were performed to investigate the associations of microbial communities with *O. sinensis* [9–14]. However, the abovementioned conclusions were drawn based on the investigation and comparison of the diversity of microorganisms between geographic regions or different tissues. Thus far, the dynamic variations in microorganisms in the life cycle of the genus *Ophiocordyceps* remain unclear.

A major challenge to this study is the limited resources available to *Ophiocordyceps* and its habitat, which includes harsh and frozen niches throughout its life cycle. *Ophiocordyceps highlandensis* [15], belonging to the same genus as *O. sinensis*, has been detected in southwestern China. It grows on larvae of Scarabaeoidea. Moreover, considerable medicinal bioactive ingredients have been detected in *O. highlandensis*. However, it has not been cultured thus far, probably due to lack of microorganisms associated with its growth. *Ophiocordyceps highlandensis* is an ideal model system for systematically comparing microbiomes at different time points due to the convenience of sampling this species at different development stages. Such a system can also be employed to explore potential core microorganisms that facilitate primordium induction and fruiting body development.

This study hypothesized that the distribution and metagenomic signatures of microorganisms change dynamically with host selection pressure. Sampling was performed in situ during *O. highlandensis* maturation. A total of 72 representative samples were obtained. High-throughput 16S and ITS2 amplicon sequencing technology were implemented to provide insights into the microbiome of *O. highlandensis* sampled at different stages of development. The present study aimed to address the following three questions: (i) How do the microbial assemblages of *O. highlandensis* vary at different developmental stages? (ii) How does the functional composition of microbial communities differ during every growth phase? (iii) Which microbial communities are associated with the abovementioned functions and what are the core groups? To the best of our knowledge, this is the first study to explore the assembly and functional dynamics of bacteria and fungi during different growth phases of *Ophiocordyceps* and to adopt network analysis tools to analyze the correlation between function and microbial communities. The results can significantly improve the facilitation of *Ophiocordyceps* cultivation*,* particularly for *O. sinensis,* and enhance the development of strategies for identifying potential bioinoculant resources.

## Results

### Bacterial community profile

**Fig. 1:**
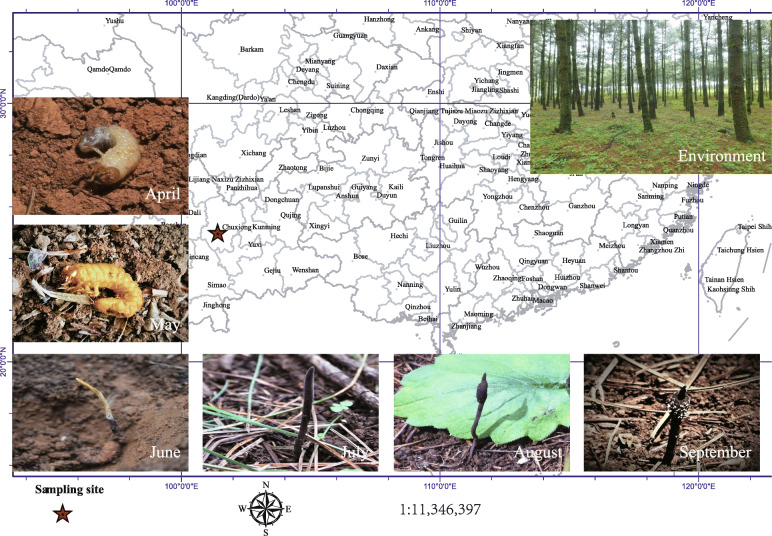
Habitats and morphology of *O. highlandensis* at six time points from April to September. The map was created with ArcGIS 10.2 software. All the photographs were generated with a regular digital camera. April, white larva; May, dead ossified larva, gold; June, stroma rising from the head of the host larva, solitary, 2–4 cm long, stipe cylindrical, 2–3 mm in diam, smooth; July, 6–8 cm long, 1.5–2.5 mm in diam, dark-brown to blackish; August, fertile portion of stroma growing; September, *Polycephalomyces synnemata* rising from the stroma of *Ophiocordyceps highlandensis* (repeated parasitization)

A total of 72 samples of *O. highlandensis* were harvested at six time points from April to September (Fig. 1). The abbreviated sample names in this study consist of locations, bacteria or fungi, and sampling time. All the samples were classified into the fruiting body (abbreviated as “cor”) and soil surrounding *O. highlandensis* (abbreviated as “soi”). During each stage, at least six samples of *O. highlandensis* were randomly collected and labeled “r1” to “r6”. For instance, sample “corF4r1” is a fungal sample from the fruiting body collected in April.

A perfect unbiased profiling procedure to illustrate the shifts in relative abundance and diversity of bacterial communities was presented based on 16 rRNA gene amplicons generated from Illumina MiSeq sequencing. A total of 5,304,053 quality-controlled sequences of the 16S rRNA gene were obtained from the bacteria in the 72 samples (Additional file 7: Table S1; Additional file 7: Table S2), with an average of 73,667 gene fragments in each sample. The rarefaction curves (Additional file 2: Fig. S2A) suggest that the number of individuals analyzed provides a reasonable representation of the diversity of the bacteria in the 72 samples. To avoid biases in sequencing depths among the samples, 36,335 sequences were subsampled randomly from each sample. Discarding low-abundance OTUs (abundance less than 0.01% in at least one sample), all the reads were clustered into 4219 OTUs with a threshold value of 97%.

**Fig. 2:**
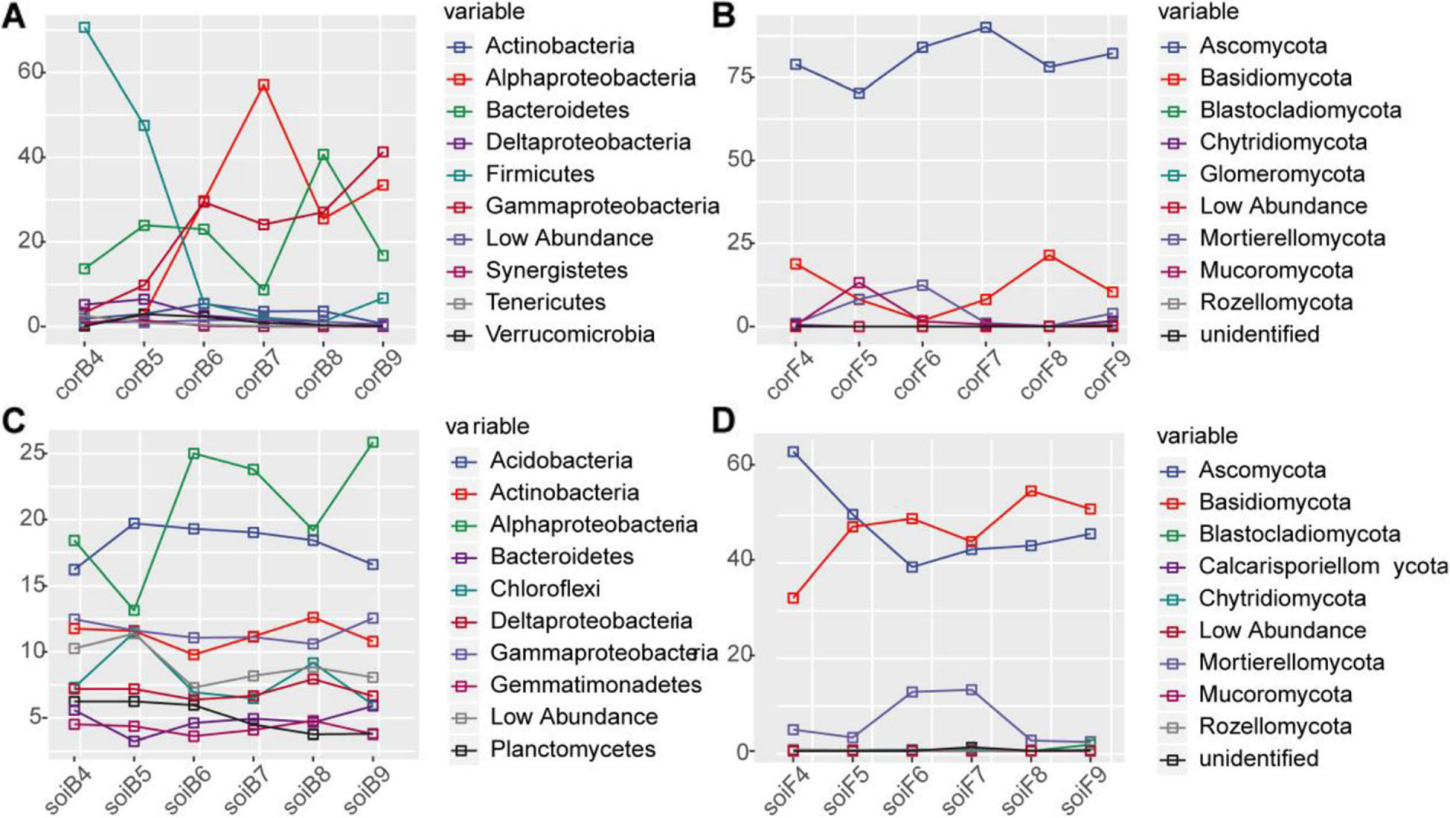
Taxonomic profiles of bacteria and fungi inhabiting *O. highlandensis* and its surrounding soil for the different developmental stages of the fungus. **A** Line chart highlighting the relative abundances of bacteria at the phylum level and class level (pertaining to Proteobacteria) in the fruiting body. **B** The relative abundances of fungi at the phylum level sampled in the fruiting body. **C** The relative abundances of bacteria at the phylum level and class level (pertaining to Proteobacteria) in the soil surrounding the fruiting body. **D** The relative abundances of fungi at the phylum level sampled in the soil surrounding the fruiting body

At the phylum and class (pertaining to Proteobacteria) levels, the dominant bacteria were also analyzed (Fig. 2A; Fig. 2C; Additional file 10: Table S9; Additional file 10: Table S10). Alphaproteobacteria was the dominant bacterial group among the fruiting body samples, accounting for 0.6–57.2% of the total reads in the samples, followed by Gammaproteobacteria (3.3–41.3%), and Firmicutes (1.2–70.7%) (Additional file 10: Table S9). The particular taxa identified in *O. highlandensis* varied with the developmental period. The relative abundances of Firmicutes, Verrucomicrobia, and Deltaproteobacteria decreased significantly during the life cycle of *O. highlandensis*, while those of Alphaproteobacteria and Gammaproteobacteria, belonging to the Proteobacteria, increased significantly (Fig. 2A). As shown in Fig. 2C and Additional file 10: Table S10, Alphaproteobacteria (13.1–25.9%) was the dominant bacteria in the soil samples, followed by Acidobacteria (16.2–19.7%) and Gammaproteobacteria (10.6–12.6%). However, variations in the soil bacteria showed a consistent trend at each developmental stage (Fig. 2C). The results suggest that the migration of bacterial flora in *O. highlandensis* is affected by developmental stage.

**Fig. 3:**
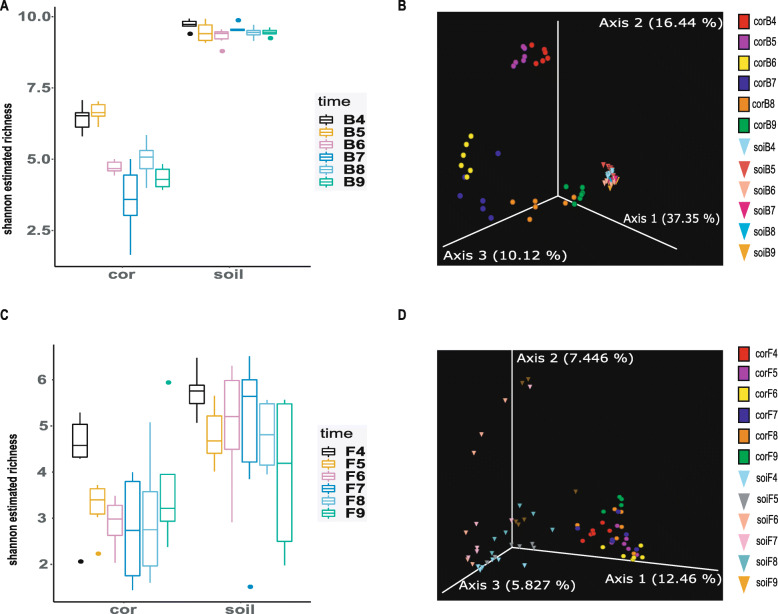
Box plot of the multivariate alpha dispersions and principal component analysis (PCA) of the bacterial and fungal composition in samples from both the fruiting body and its surrounding soil. **A** Comparisons of bacterial alpha dispersions (Shannon richness index) grouped with multivariate variables. **B** PCA of bacteria based on a Bray-Curtis distance matrix. **C** Comparisons of fungal alpha dispersions (Shannon richness index) grouped with multivariate variables. **D** PCA of fungi based on a Bray-Curtis distance matrix

In the analysis of the alpha diversity (Additional file 7: Table S1; Additional file 2: Table S2) of *O. highlandensis*-inhabiting bacteria, both the Shannon and Evenness diversity parameters had relatively high volatility in each categorical group (Fig. 3A; Additional file 3: Fig. S3A). It is noteworthy that the results of this analysis in the soil samples did not different within each categorical group (Fig. 3A; Additional file 3: Fig. S3A). The Kruskal-Wallis test showed significant differences in the Shannon diversity and Evenness categorized by the developmental stage of the fruiting body (Table 1; *P* < 0.001). The results suggest slightly significant differences in the soil samples (Table 1; *P* = 0.152 and *P* = 0.093). Moreover, a pairwise Kruskal-Wallis test also suggested significant divergence among the *O. highlandensis*-inhabiting bacterial community (Table 2; *P* < 0.05 or *P* < 0.01). However, the results from the soil samples were not similar (*P* > 0.05) (Additional file 8: Table S5). These results are consistent with the pairwise Permutational MANOVA test on Beta diversity dispersion analysis. (*P* < 0.05) (Additional file 9: Table S8).

**Table 1:** Results of a Kruskal-Wallis test of the bacterial and fungal community composition in the fruiting body and microhabitat soil samples. H is the statistical parameter for Kruskal-Wallis tests. The *P*-values are based on the Shannon diversity and Evenness indexes computed from the OTU tables. The bold values indicate statistically significant results

		Bacteria		Fungi	
		H	*P*-value	H	*P*-value
Fruiting Body	Shannon diversity	26.910	< 0.01	6.918	0.227
	Evenness	27.933	< 0.01	7.055	0.217
Microhabitat Soil	Shannon diversity	8.07	0.152	6.907	0.228
	Evenness	9.440	0.093	7.601	0.180

**Table 2:** Pairwise comparisons of the Shannon diversity and Evenness indexes of the bacterial groups inhabiting the fruiting body. The bold values indicate statistically significant results

shannon	corB4	corB5	corB6	corB7	corB8	corB9
Evenness						
corB4		0.565	0.007	0.007	0.010	0.007
corB5	0.289		0.007	0.007	0.007	0.007
corB6	0.007	0.007		0.134	0.299	0.134
corB7	0.007	0.007	0.052		0.036	0.233
corB8	0.007	0.007	0.556	0.023		0.075
corB9	0.007	0.007	0.072	0.229	0.072	

Variations in the bacterial communities were explored by principal component analysis (PCA) of all the samples (Fig. 3B). The PCA results indicated that at the OUT level, the bacterial assemblages from the fruiting body and surrounding soil can be significantly parsed into two major subgroups, indicating that there are significant differences in the total community structures between *O. highlandensis* and the corresponding soil environments. Note that the bacterial samples from *O. highlandensis* were clustered together based on the different developmental stages sampled (Fig. 3B). However, compact clustering of all 36 soil samples was observed in the bacterial communities (Fig. 3B). These results suggest that there are different bacteria in *O. highlandensis* at different developmental stages. The patterns of the bacterial communities in *O. highlandensis* may be defined by the developmental phase of this fungus.

### Fungal community richness and diversity

A total of 5,944,256 raw sequences of the ITS2 gene were obtained from the fungi in the 72 samples. After quality-control steps, approximately 4,693,765 sequences were retained for subsequent analyses (Additional file 7: Table S3; Additional file 7: Table S4). The rarefaction curves for the observed ASVs revealed that the number of individuals analyzed adequately represented the diversity of the fungi in the 72 samples (Additional file 2: Fig. S2B). To avoid biases from the different sequencing depths among the samples, 22,980 sequences were randomly subsampled from each sample. After the low-abundance ASVs (abundance less than 0.01% in at least one sample) were discarded, all the reads were clustered into 3558 ASV units.

The fungal community structure was also analyzed. At the phylum level, among the fruiting body samples, the greatest number of sequences belonged to Ascomycota (Fig. 2B; Additional file 10: Table S11), accounting for 70.3–90.1% of the total reads in the respective samples, followed by Basidiomycota (1.8–21.6%) and Mortierellomycota (0.2–12.5%). No apparent change was identified in the particular taxa related to *O. highlandensis* with developmental period (Fig. 2B). Ascomycota (38.5–62.8%) was the dominant phylum in the soil samples, followed by Basidiomycota (32.1–54.5%) and Mortierellomycota (1.9–12.8%) (Fig. 2D; Additional file 10: Table S12). Furthermore, variations in the soil fungal community displayed consistent trends at each developmental stage (Fig. 2D). These results showed that the fungal community structure was not affected by developmental stage in both *O. highlandensis* and its surrounding soil.

When analyzing alpha diversity (Additional file 7: Table S3; Additional file 7: Table S4), neither the Shannon diversity nor the Evenness indexes exhibited relatively high volatility in any of the samples of *O. highlandensis* or its surrounding soil (Fig. 3C and Additional file 3: Fig. S3B). The Kruskal-Wallis test suggested no significant differences when the data were categorized by developmental stage in either the fruiting body or its surrounding soil (Table 1; *P* > 0.05). Moreover, a pairwise Kruskal-Wallis test suggested that the fungal community sampled at specific time points was similar to that in the two environments (P > 0.05) (Additional file 8: Table S6; Additional file 8: Table S7).

Principal component analysis (PCA) and beta dispersion analyses revealed that fungal assemblages from the fruiting body and its surrounding soil can be parsed significantly into two major subgroups as well. For this reason, it is suggested that there are significant differences in the overall fungal community structures between *O. highlandensis* and its corresponding soil environments. However, the fungi did not cluster together according to different developmental stages for the samples from either *O. highlandensis* or the soil (Fig. 3D). These results suggest that the developmental stage of *O. highlandensis* may slightly impact the observed fungal ASV composition.

### Diversity of bacterial communities among development stages of *O. highlandensis*

**Fig. 4:**
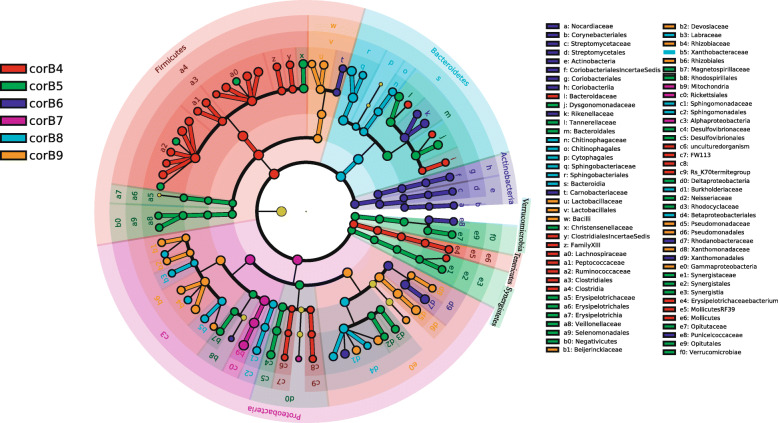
LEfSe analysis (LDA) of the bacterial taxa (LDA score > 3.5) characterizing the differences in the phylogenetic richness among samples. The circles indicate the taxonomic level (from phylum to genus). The letter and number combinations indicate different taxonomic groups (from order to family). The size of the circles indicates the abundance of each community. Different group regions are colored uniquely

Compared with the fungi, an unexpectedly high taxonomic diversity was observed in the bacterial community during the maturation of *O. highlandensis*. We aimed to identify particular bacterial communities among the samples from different developmental stages. Thus, linear discriminant analysis (LDA) was executed with the LEfSe tool. Only LDA scores of 3.5 or higher are shown in the cladograms (Fig. 4; Additional file 4: Fig. S4). Four bacterial groups were significantly enriched in the corB4 samples (Fig. 4) (a family and three genera in the order Bacteroidales): Firmicutes (from phylum to genus), Tenericutes (from phylum to genus), a genus in the family Synergistaceae, and two orders (the order and its family and genus) in the Proteobacteria. However, in the corB5 samples, five bacterial groups were significantly enriched: Bacteroidales (the order and its family and genus), Christensenellaceae (the family and its genus) and three genera in Clostridiales, two classes (from class to genus) belonging to the Firmicutes, two phyla (from phylum to genus), Deltaproteobacteria (from class to genus) and Rhodospirillales (the order and its family and genus) and two families (the family and genus). CorB6 primarily included five groups: Actinobacteria (from phylum to genus), Rikenellaceae (the family and its two genera), Carnobacteriaceae (the family and its genus), two genera and Xanthomonadales (from order to genus), and Puniceicoccaceae (the family and its genus). CorB7 was enriched with Proteobacteria (from phylum to genus). CorB8 was significantly enriched with two bacterial groups: Bacteroidetes (from phylum to genus), two orders (from order to genus) and two families (the family and its genus) as well as two genera. CorB9 included two enriched bacterial groups, namely, Bacilli (from class to genus), belonging to the Firmicutes, and Gammaproteobacteria (from class to genus) and Rhizobiales (from order to genus), belonging to the Proteobacteria.

### Assessment of function potentials

**Fig. 5:**
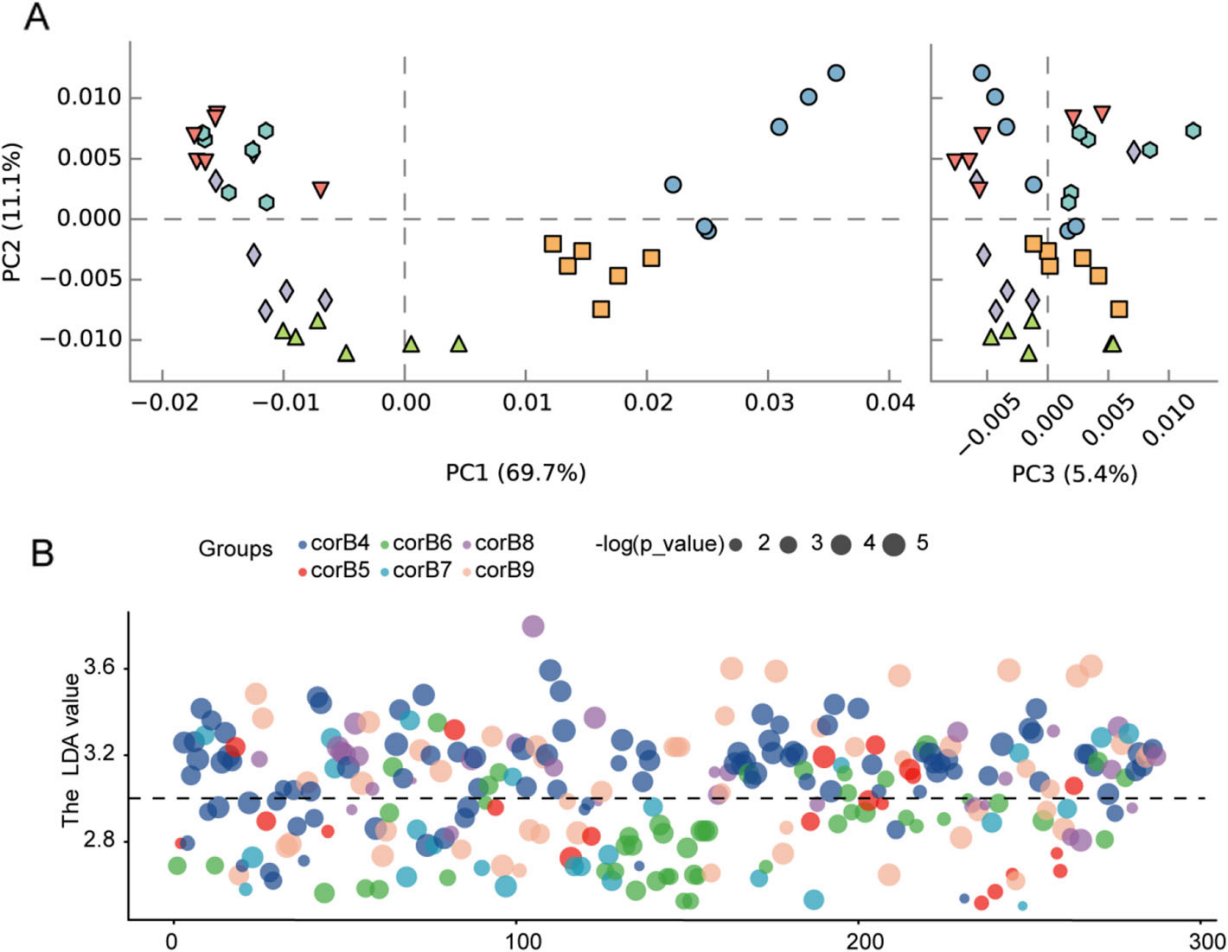
Different functions among the samples. **A** Principal coordinate analysis of the functional pathways represented by the endogenic bacterial communities. The analysis relies on a Bray-Curtis distance matrix and shows the percentage of each axis on the graph explaining the total variance. **B** Different pathways represented by *O. highlandensis*-inhabiting bacterial communities in the developmental stages analyzed. A *P*-value (log 10) higher than three was considered significant

PICRUSt2 was applied to assess the functional profiles of the bacterial communities inhabiting *O. highlandensis* with a computational approach. The weighted nearest sequenced taxon index (NSTI) value distribution was largely less than 0.2 (Additional file 5: Fig. S5), proving that the results of the functionality assessment were accurate. Samples of *O. highlandensis* were aggregated into different clusters according to the developmental stages (Fig. 5A). These results suggest that the relative abundances of the functional profiles are largely different at each sampling time (Fig. 5B; Additional file 11: Table S13). Among the *Ophiocordyceps highlandensis*-inhabiting bacteria, carbohydrate degradation pathways were significantly abundant in the corB4 samples. There were more de novo nucleotide biosynthesis pathways in corB5. A group of pathways associated with the biosyntheses of pyridoxal and lipids were enriched in the corB6 samples. In addition, the pathways of the TCA cycle (tricarboxylic acid cycle) in the corB7 samples were rather abundant. In the corB8 samples, there were some dominant pathways associated with amino acid biosynthesis. Moreover, pathways related to the biosynthesis of mycolate, oleate and palmitoleate were significantly more abundant in the corB9 samples. These findings confirm that *O. highlandensis* developmental stage determines the functional pattern of its associated bacteria.

### The bipartite network analysis

**Fig. 6:**
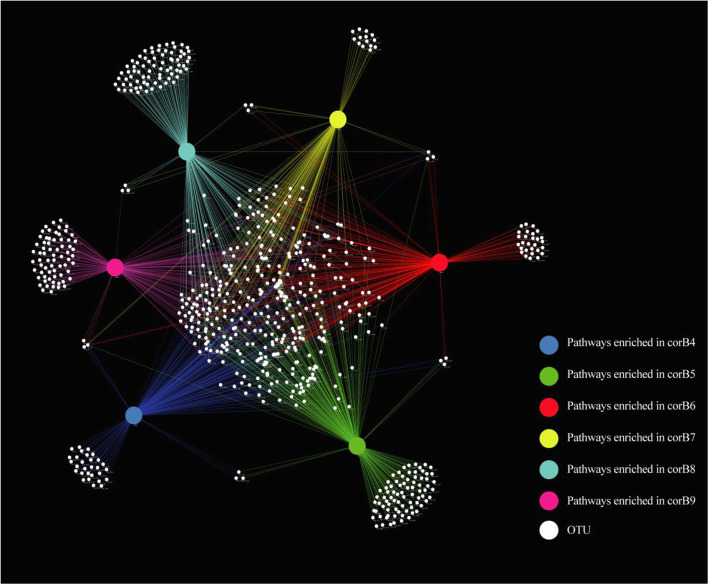
The bipartite network based on the relationships between OTUs and pathways enriched in six different developmental stages of *O. highlandensis*. Each significantly enriched pathway in the different stages is marked with large colored circles (pathway_nodes). The small circles (OTU_nodes) represent the OTUs, shown in white. The edges connect through the OTU_nodes to the pathway_nodes in which they are included. The inner OTU nodes connect multiple pathway nodes, while the unique OTU nodes in each period are located around the outside of the bipartite network

To identify the core microbes, a connected network was built, characterizing which microorganisms contributed to the different functions in each developmental period (Fig. 6). The bipartite network consisted of six large nodes (pathway_nodes), representing special metabolic pathways at six different times, and 678 OTU_nodes. These six pathway nodes contained a considerable number of unique OTU nodes. The edges were connected via each OTU_node to the pathway_nodes, where they were included uniquely. The topological analysis of the bipartite network revealed that two prominent hubs (Pathway_corB4 and Pathway_corB5) had extremely high numbers of unique OTUs, while the Pathway_corB7 hubs showed the smallest number of unique OTUs (Fig. 6). Furthermore, the degree of network distribution revealed that just four OTU_nodes are shared in all six developmental stages. These OTUs may impact critical functions during different growth periods of *O. highlandensis*. They pertain to the family Xanthobacteraceae and the genera *Pseudomonas*, *Bradyrhizobium* and *Mycobacterium* (Additional file 12: Table S14). In brief, the network perspective revealed the complex structure of the relationships between OTUs and pathways, and four potential core OTUs were clearly identified.

## Discussion

### Fluctuation in bacterial community during *O. highlandensis* fruiting body development

Most members of the genus *Ophiocordyceps* have been demonstrated to be very valuable in traditional medicines in China. However, the increasing social demand and the decreasing supply of natural *Ophiocordyceps* may soon lead to its extinction. Many previous studies have primarily focused on environmental conditions or genetic features associated with *Ophiocordyceps* to resolve issues with and improve cultivation yields [16–19]. However, little progress has been made to date. Bioinoculants comprising some beneficial microbes have been reported to be capable of stimulating the growth and production of mushrooms [20, 21]. Thus far, the microbial composition associated with the genus *Ophiocordyceps* at each developmental stage has remained unclear. The major challenge to the study of this topic is likely the limited number of *Ophiocordyceps* and harsh conditions in which it grows throughout its life cycle. In the present study, we found that uncultured *O. highlandensis* can act as a suitable model. Sampling was performed in this study during different stages of naturally occurring *O. highlandensis*, and relative estimates of the microbial diversity in *O. highlandensis* and its soil microhabitat were obtained. The results of this study reveal that the community structure of *O. highlandensis*–inhabiting bacteria varies dramatically across the developmental stages of this species (Fig. 3), while the structure of the fungi undergoes relatively minor variations (Fig. 3). Notably, the presence of *Polycephalomyces agaricus*, observed in our previous study [22], did not lead to alterations in the endogenous fungal diversity. The bacteria in the soil, which served as a control, were mostly unchanged. Moreover, a systematic comparative study showed functional diversity in the bacterial community during *O. highlandensis* maturation. All these results indicate that *O. highlandensis* may recruit different endogenous bacteria throughout its life cycle to enhance its growth and support rapid infection.

Numerous researchers have studied the rich diversity of many endogenic *Ophiocordyceps*-inhabiting microbial communities [7, 9–14, 23, 24]. These studies suggest that species belonging to the Ascomycota are the most abundant in the fruiting body and soil microhabitats, which is consistent with our results (taking up 70.3–90.1%). In this study, the majority of bacteria observed in *O. highlandensis* belonged to Proteobacteria and Firmicutes, while Proteobacteria and Acidobacteria were found in the soil microhabitat. This is consistent with some previous studies [12] and contrary to others [11, 13]. This discrepancy can be explained by our finding that bacteria in *O. highlandensis* fluctuate more strongly than fungi during different developmental stages. Moreover, in the investigation of microorganisms inhabiting macrofungi, it is important to consider the same sampling time.

Throughout the life cycle of *O. highlandensis*, the relative abundance of Proteobacteria increased significantly (Fig. 2A). This phenomenon was also observed when assessing the composition of the microbiome in cultivated *A. bisporus* [25]. The presence of *O. highlandensis* considerably enhances the growth of Proteobacteria as fruiting body maturation progresses, thus indicating a synergistic interaction between the Proteobacteria phylum and *O. highlandensis*. Notably, the relative abundance of Firmicutes decreased during almost all developmental phases (Fig. 2A). For Bacteroidetes and Proteobacteria, the opposite trend (i.e., rising) occurred throughout the mature stage, indicating that there may be a relatively strong competitive relationship between these phyla. These findings support the assumption that native microbial communities can hinder the growth of competitors and play a vital role in the colonization of host fungi [26].

A considerable number of studies have reported that some Ascomycetes can shape the bacterial communities in the surrounding soil. The soil surrounding *Tuber melanosporum* exhibits distinct bacterial diversity compared with that in bulk soil [27]. Likewise, an investigation targeting *Moraxella osloensis* distribution suggests a higher abundance in areas colonized by *Tuber magnatum* [28]. Though the microbial structure of bulk soil was not investigated in *O. highlandensis’* living area, this study speculates that the observed patterns slightly support the above observations. The microbial community inhabiting the bulk soil exhibited a higher diversity index than that inhabiting the fruiting bodies (Fig. 3 and Additional file 3: Fig. S3). Both the bacterial and fungal samples of *O. highlandensis* and its surrounding soil formed two significantly separate clusters (Fig. 3B and D). These results suggest that there are distinct microbial community structures between the fruiting body and its surrounding soil. The bacterial composition in *O. highlandensis* at different development stages shows considerable clusters. However, clusters were not identified in the soil. Accordingly, during maturation, selection by naturally occurring *O. highlandensis* became weaker on the microbiome in the soil surrounding the fungus. Moreover, an existing study suggests that there is no significant difference in the bacterial diversity index between the sites where *O. sinensis* is present and sites where it is not [23]. It is logical to speculate that *O. highlandensis* cannot shape the distribution of bacteria in its surrounding soil.

### Fungal helper bacteria are enriched at different developmental stages

Fungal helper bacteria are required at different developmental stages of macrofungi [5, 6, 8, 29, 30]. In corB4, the mycelia of *O. highlandensis* start the colonization process. The bacterial community was dominated by the phylum Firmicutes. Members from this phylum have been reported to be involved in converting complex carbohydrates into monosaccharides for absorption by mycelia [25]. This is also consistent with the enrichment of the pathway associated with glycogen degradation during this stage. In corB5, the larval host is killed. A considerable number of Synergistetes members have been suggested to be significant contributors to gastrointestinal infections [31]. These bacteria may help host fungi infect and kill larvae. This is also consistent with our finding based on the LDA score that the de novo nucleotide biosynthesis pathway differs between this and other stages. These findings suggest that other microbes, with the help of Synergistetes, may reproduce rapidly in larvae. This is also reflected in the increase in the Shannon index during this period (Fig. 3A). In corB6, the stroma arises from the head of the larval host. Organic compounds from the ossified larva may be the main nutrition source for *O. highlandensis*. High proportions of Actinobacteria and subgroups from the dominant phylum Proteobacteria were observed. It has been reported that species from these taxa decompose organic compounds into available carbon sources for fungi in the differentiation phase [32, 33]. Some pathways associated with the biosynthesis of pyridoxal and lipids were enriched, revealing that many bacteria can provide growth factors for *O. highlandensis*. In corB7, the stroma grows from 2 to 4 cm to 6–8 cm. Alphaproteobacteria are dominant in this stage. This phenomenon was consistent with the peak of Alphaproteobacteria in the corB7 (Fig. 2A), also indicating that there may be an intense synergistic interaction between Proteobacteria and *O. highlandensis* during this stage. Some other macrofungi have also been reported to enhance the growth of Proteobacteria [25]. Pathways associated with the TCA cycle appeared to be more abundant in this stage. These pathways may provide a necessary source of intermediate organic matter for some biosynthesis that takes place during stroma elongation. In corB8, the fertile portion of the stroma grew. The dominant phylum was Bacteroidetes. These bacteria have been described as contributors to the degradation of chitin and cellulose [34]. The fertile portion may act as a major nutrition sources by the members in phylum Bacteroidetes now. In corB9, it was observed that *P. agaricus* was parasitic on *O. highlandensis* and grew shorter fruiting bodies (Fig. 1). *O. highlandensis* achieves high levels of genus *Pseudomonas* (Additional file 4: Fig. S4). It has been reported that species belonging to Pseudomonas are associated with beneficial interactions with mycorrhiza and fungi [35]. These species are also correlated with the induction and promotion of fructification during the process of macrofungi cultivation [33]. To the best of our knowledge, this is the most comprehensive report of specifically enriched bacteria and functions across different developmental stages of the genus *Ophiocordyceps*.

### Discovering core microbial groups

Identifying core microorganisms is critical for managing microbial assemblages and achieving desired outcomes [36]. Although several researchers have tried to identify core microbial groups that are critical to the growth of macrofungi, they all relied on Venn diagram analyses based on presence/absence datasets [12, 37]. The results are inconsistent and should be interpreted carefully. More importantly, the functions of core microorganisms are not apparent from a Venn diagram analysis. The functional composition of microorganisms can be visualized and interpreted precisely with a variety of powerful bioinformatic tools, e.g., PICRUSt1, Tax4Fun, Piphillin, PanFP and PAPRICA [34, 38–41]. PICRUSt2 was recently released. The reference genomes and gene family databases for assessing functional signatures of the 16S rRNA gene were expanded compared with those in other software programs [42]. The ability of PICRUSt2 to identify potential functions in a community was significantly enhanced.

Thus, essential microorganisms can be defined by a comparative network analysis between functions and OTUs. In total, four potential core OTUs participating in different functions during each developmental period were retrieved (Additional file 12: Table S14). OTU_5 had 100% 16S rRNA identity with *Pseudomonas* sp. isolated from the stem of *Ziziphus jujuba Mill* (according to data in GenBank). This species can enhance host growth. OTU_9 exhibits 100% sequence identity with *Bradyrhizobium* sp*.* isolated from a root nodule of *Lespedeza cuneata*. OTU_144 had 100% 16S rRNA identity with *Mycolicibacterium madagascariense* isolated from *Sphagnum obtusiusculum* [43]*.* However, isolating the core OTUs by metagenome study is required to further verify whether they are associated with the growth of genus *Ophiocordyceps* in our subsequent research.

## Conclusions

All the findings of this study suggest an unexpectedly high taxonomic and functional fluctuation in the bacterial community during *O. highlandensis* maturation. Moreover, four potential core OTUs were identified. Our subsequent research aims to gain insights into intriguing genetic mechanisms by metagenomic and metatranscription analysis. We believe that our findings offer useful insights for the cultivation of members of *Ophiocordyceps*, including *O. sinensis*.

## Methods

### Sampling procedure

*Ophiocordyceps highlandensis* samples were obtained at six different time points from April–September 2018 during its development in the Songming region (Fig. 1). During each stage, at least six sample sites were randomly selected from a 10 m × 10 m quadrat. Both *O. highlandensis* and its surrounding soil were collected. The surface putrilage on each soil sample was removed, and approximately 15 g of the surrounding soil was collected with a sterile shovel. Then, all samples were packed into a 50 ml sterile Eppendorf tube and transferred on ice to the laboratory for subsequent analyses. The fruiting body was sterilized with 75% ethanol for 2–3 min, then with 30% hydrogen peroxide for 3–5 min and subsequently washed with sterile water three times [23]. All sampling distribution is illustrated clearly in Fig. 1. The values of the environmental parameters at each site, including the total nitrogen (g/kg), total phosphorus (g/kg), total potassium (g/kg), total carbon (g/kg), dissolved organic carbon (g/kg), C/N, humidity (%), and pH, are shown in Additional file 1: Fig. S1.

### DNA extraction and PCR amplification

In total, we obtained 72 samples of *O. highlandensis* (36 samples) and its surrounding soil (36 samples). To enhance the DNA yield, each *O. highlandensis* tissue sample was fully ground and mixed in liquid nitrogen. Next, total DNA was extracted immediately with a MicroElute Genomic DNA Kit (http://www.annoron.com/) in accordance with the manufacturer’s manual. The DNA of soil sample (about 400 mg per soil sample) was extracted with a PowerSoil DNA Isolation Kit (Mo Bio Laboratories, USA). The purity and quantification of the extracted DNA was determined with a NanoDrop 2000 UV-Vis spectrophotometer (Thermo Scientific, Wilmington, USA). The genomic DNA was frozen at − 80 °C until it was used for subsequent analysis.

### PCR amplification

The hypervariable V3-V4 region of the 16S rRNA gene fragment was PCR (polymerase chain reaction) amplified with the universal primer set 341F (5′ -CCTAYGGGRBGCASCAG-3′) and 806R (5′- GGACTACNNGGGTATCTAAT -3′) [44]. For the fungal community analysis, ITS2 gene fragments were PCR amplified with the universal primer set ITS3-2024F (5′ - GCATCGATGAAGAACGCAGC − 3′) and ITS4-2409R (5′- TCCTCCGCTTATTGATATGC -3′) [45]. Six barcode nucleotides were attached to the primers to distinguish the respective samples. PCRs were performed in triplicate with 30 μL of PCR mixture supplemented with 15 μL of Phusion® High-Fidelity PCR Master Mix (New England Biolabs), 0.2 μM of each primer and 10 ng of template DNA. The thermal cycling program included initial denaturation at 98 °C for 1 min; 30 cycles of 10 s at 98 °C, 30 s at 50 °C, 30 s at 72 °C; and a final elongation step at 72 °C for 5 min.

### Sequencing

The PCRs were performed in triplicate for each sample. To ensure the homogeneity of the PCR process, the products for each sample were pooled together and checked with agarose gel electrophoresis (Additional file 6: Fig. S6-S9). Subsequently, the relevant PCR fragments were extracted from the agarose gel and further purified with a GeneJET Kit (Thermo Scientific). The PCR product concentration was quantified with QuantiFluor™ ST (Promega, USA) according to the manufacturer’s instructions. The purified DNA from the respective samples was pooled equimolarly. In total, we obtained 144 sequencing libraries (including 72 16S-rRNA-gene sequencing libraries and 72 ITS2 sequencing libraries). An Ion Plus Fragment Library Kit 48 rxns (Thermo Scientific) was employed to generate 16S rRNA gene sequencing libraries. Finally, the libraries were sequenced on an Ion S5TM XL platform. ITS2 gene fragment sequencing was conducted on an Illumina MiSeq platform (Illumina, San Diego, USA) following an optimization protocol described by Majorbio Bio-Pharm Technology Co. Ltd. The total raw reads were deposited in the NCBI Sequence Read Archive (SRA) database (accession numbers SRR10550041 to SRR10550184).

### Sequencing data processing

The 144 raw fastq files were imported into QIIME2 version 2019.4 [46] (based on the tutorial https://docs.qiime2.org/2019.4/tutorials/) and subsequently demultiplexed with the package ‘q2-demux’ version 2019.4.1. The Illumina adaptors and amplification primers were removed with package ‘q2-cutadapt’ version 2019.4.0. Quality filtering, denoising, and merging of paired sequences were performed in the DADA2 pipeline [47]. After this process, a higher resolution amplicon sequence variant (ASV) table was generated for the fungi in the samples, and an operational taxonomic unit (OTU) table based on 16S rRNA genes clustered at 97% similarities was generated for the bacteria. Next, the OTU table was filtered to retain those with expected relative abundances over 0.01%. The taxonomy of each representative sequence was assigned with a classifier before being trained on the SILVA reference database (for the bacteria) or the QIIME reference dataset released by UNITE (for the fungi). To fit the analytical bias, the sequences in the individual samples were randomly subsampled to an equal number. Alpha and beta diversity parameters were computed based on a phylogenetic tree, and rarefied OTU tables were generated through QIIME 2’s diversity plugin.

### Metagenome imputation from 16S rRNA datasets

PICRUSt2 was adopted to assess the potential functional profiles of the bacterial metagenomes in *O. highlandensis* from the 16S rRNA data [42]. The OTU table was mapped to an updated database of reference genomes and gene families. The 16S rRNA gene copy number was normalized based on the Genome Prediction Tutorial for PICRUSt2. The metagenomes were assessed against the MetaCyc database of functional pathways. Statistical visualization and analyses were performed for all the results.

### Statistical analysis

Line charts and box plots were created with the “gplot2” package in R. The significance of the alpha diversity among different stages was assessed by Kruskal-Wallis tests in all groups, and pairwise tests were performed by the Benjamini-Hochberg FDR method with test correction. Redundancy analysis (RDA) was conducted with the LEfSe tool [48]. The bacterial OTUs and fungal ASV sequence counts were summarized at the phylum level with R.

### Detection and visualization of supercore microorganisms

To ascertain the distinct contribution of OTUs to special functions, a bipartite network was reconstructed with established methods. In brief, the functional pathways explicitly enriched in each period were identified. Only OTUs present in the mentioned pathways and appearing in all the samples underwent a downstream analysis in the corresponding group. These processes were carried out in Python 3 and R. make_bipartite_network.py was employed to build the network. Next, the final network files, including nodes and edges, were edited and displayed with Cytoscape software (version 3.3.0). Next, a visualization of the network, with an edge-weighted spring-embedded layout, was created.

## Supplementary Information

**Additional file 1: Fig. S1**. Abiotic environmental variation in the *O. highlandensis* habitats featured in this study. The TN (total nitrogen), TP (total phosphorus), TK (total potassium), TC (total carbon), DOC (dissolved organic carbon), C/N (carbon-nitrogen ratio), humidity (%), and pH of each sampling site are shown.

**Additional file 2: Fig. S2**. Rarefaction curves of the bacterial (A) and fungal (B) communities collected from fruiting bodies of *O. highlandensis* and the surrounding soil.

**Additional file 3: Fig. S3**. Box plot of multivariate alpha dispersions of the microbial composition. (A) Comparisons of the bacterial alpha dispersions (Evenness parameter) grouped with multivariate variables. (B) Comparisons of the fungal alpha dispersions (Evenness parameter) grouped with multivariate variables.

**Additional file 4: Fig. S4**. Distinct taxonomic composition among groups during *O. highlandensis* maturation. An LDA score (−log 10) above 3.5 was considered statistically significant.

**Additional file 5: Fig. S5**. Density plots of the NSTI values from the function prediction process performed with PICRUSt2 software.

**Additional file 6: Fig. S6**. Gel image for DNA extracted from soil; numbers 1–6 were collected in April and 14–19 in May. A and B were original gel image, the B was the second test result of sample 6 and 16, the sample and all procedures were same with the first test. C was processed in Power Point, numbers 1–5,14-15,17–19 of C were from A. numbers 6,16 of C were from B. **Fig. S7**. Gel image for DNA extracted from soil; numbers 1–6 were collected in June and 7–12 in July. A and B were original gel image, the B was the second test result of sample 4, the sample and all procedures were same with the first test. C was processed in Power Point, numbers 1–3,5–12 of C were from A, numbers 4 of C was from B. **Fig. S8**. Gel image for DNA extracted from soil; numbers 1–6 were collected in August. **Fig. S9**. Gel image for DNA extracted from soil; numbers 7–12 were collected in September. A was original gel image, B was processed in Power Point, numbers 7–12 of B were from A.

**Additional file 7: Table S1**. Summary of the sequences and richness and diversity indexes of the bacteria in the fruiting bodies. **Table S2**. Summary of the sequences and richness and diversity indexes of the bacteria in the soil microhabitat. **Table S3**. Summary of the sequences and richness and diversity indexes of the fungi in the fruiting bodies. **Table S4**. Summary of the sequences and richness and diversity indexes the fungi in the soil microhabitat.

**Additional file 8: Table S5**. Pairwise comparisons of the Shannon index and Evenness parameter among the bacterial groups inhabiting the soil microhabitat. The bold values represent statistically significant results. **Table S6**. Pairwise comparisons of the Shannon index and evenness parameter among the fungal groups inhabiting the fruiting body. The bold values indicate statistically significant results. **Table S7**. Pairwise comparisons of the Shannon index and Evenness parameter among the fungal groups inhabiting the soil microhabitat. The bold values represent statistically significant results.

**Additional file 9: Table S8**. Results of a pairwise permutational MANOVA on the beta diversity of the bacterial community composition in the fruiting body. ​*P*-values result from 999 permutations of a Bray-Curtis dissimilarity matrix. The bold values indicate statistically significant results.

**Additional file 10: Table S9**. Relative abundances of the bacterial phyla and classes belonging to the Proteobacteria in all the fruiting body samples. **Table S10**. The relative abundances of the bacterial phyla and classes belonging to the Proteobacteria in all the soil microhabitat samples. **Table S11**. The relative abundances of the fungal phyla in all the fruiting body samples. **Table S12**. The relative abundances of the fungal phyla in all the soil microhabitat samples.

**Additional file 11: Table S13**. Distribution of the distinct bacterial functions during *O. highlandensis* maturation.

**Additional file 12: Table S14**. Taxonomic summary of 4 supercore microorganisms.

## Data Availability

All the high-throughput sequencing data were deposited in the National Center for Biotechnology Information (NCBI) database under project number PRJNA592049 (link: https://www.ncbi.nlm.nih.gov/sra?linkname=bioproject_sra_all&from_uid=592049).

## Abbreviations

PCR: Polymerase chain reaction
OTU: Operational taxonomic unit
ASV: Amplicon sequence variant
RDA: Redundancy analysis
PCA: Principal component analysis
LDA: Linear discriminant analysis
TCA cycle: Tricarboxylic acid cycle
PICRUSt2: Phylogenetic communities by reconstruction of unobserved states 2
TN: Total nitrogen
TP: Total phosphorus
TK: Total potassium
TC: Total carbon
DOC: Dissolved organic carbon
C/N: Carbon-nitrogen ratio
