# Supplementary material for: Endogenous bacteria inhabiting the Ophiocordyceps highlandensis during fruiting body development
Source: BMC Microbiol. 2021 Jun 11;21:178. doi: 10.1186/s12866-021-02227-w (PMC8196446; doi:10.1186/s12866-021-02227-w)
Supplement: Supplementary file 1 — Additional file 1: Fig. S1. Abiotic environmental variation in the O. highlandensis habitats featured in this study. The TN (total nitrogen), TP (total phosphorus), TK (total potassium), TC (total carbon), DOC (dissolved organic carbon), C/N (carbon-nitrogen ratio), humidity (%), and pH of each sampling site are shown. [file 12866_2021_2227_MOESM1_ESM.docx]

Endogenous bacteria inhabiting the *Ophiocordyceps highlandensis* during fruiting body development

Chengpeng Li^2#^, Dexiang Tang^1,2#^, Yuanbing Wang^1,3^, Qi Fan^1^, Xiaomei Zhang^1,3,4^, Xiaolong Cui^2*^ and Hong Yu^1*^


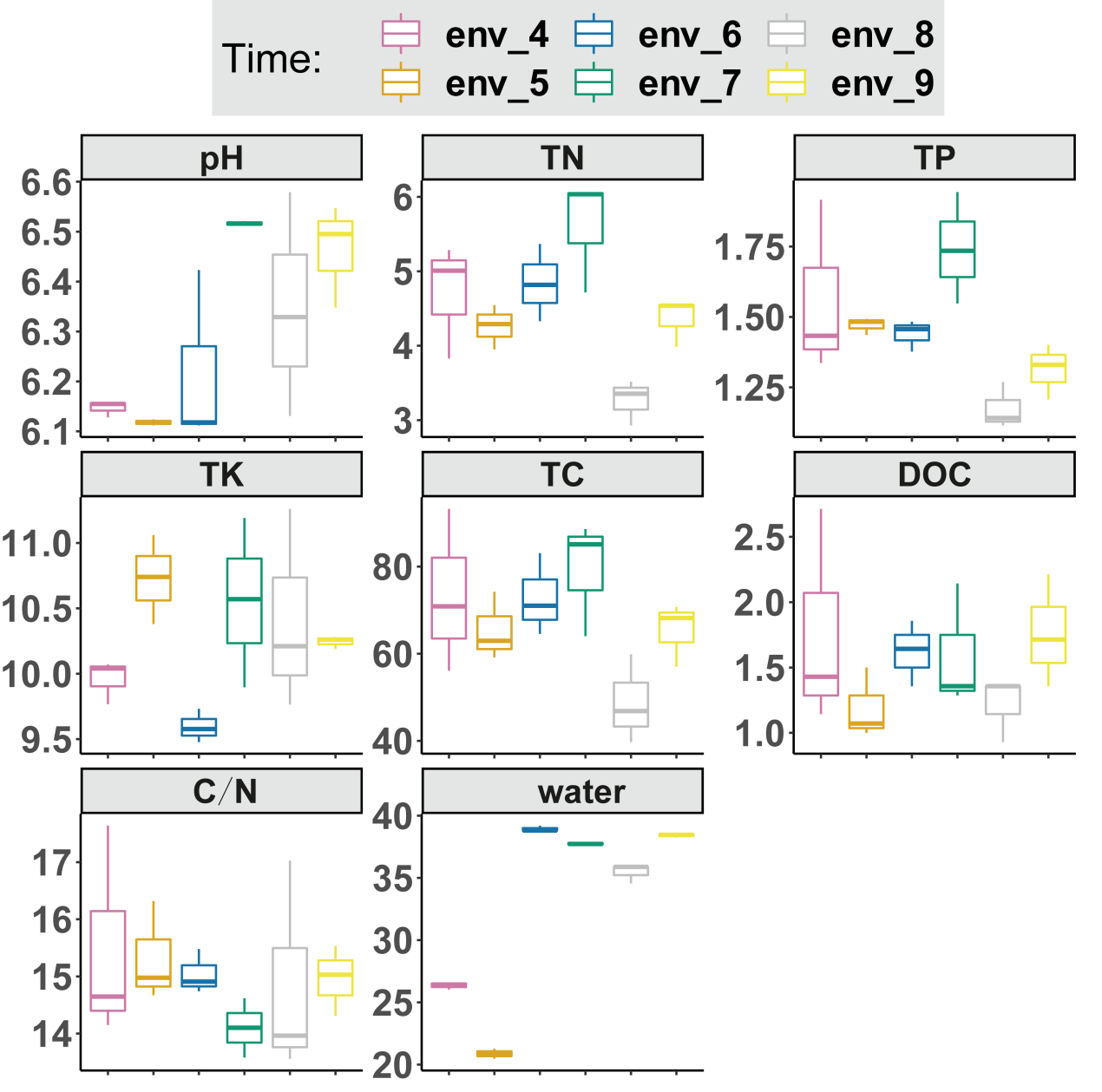


Additional file 1: Fig. S1. Abiotic environmental variation in the O. highlandensis habitats featured in this study. The TN (total nitrogen), TP (total phosphorus), TK (total potassium), TC (total carbon), DOC (dissolved organic carbon), C/N (carbon-nitrogen ratio), humidity (%), and pH of each sampling site are shown..
