# Supplementary material for: Endogenous bacteria inhabiting the Ophiocordyceps highlandensis during fruiting body development
Source: BMC Microbiol. 2021 Jun 11;21:178. doi: 10.1186/s12866-021-02227-w (PMC8196446; doi:10.1186/s12866-021-02227-w)
Supplement: Supplementary file 2 — Additional file 2: Fig. S2. Rarefaction curves of the bacterial (A) and fungal (B) communities collected from fruiting bodies of O. highlandensis and the surrounding soil. [file 12866_2021_2227_MOESM2_ESM.docx]

Endogenous bacteria inhabiting the *Ophiocordyceps highlandensis* during fruiting body development

Chengpeng Li^2#^, Dexiang Tang^1,2#^, Yuanbing Wang^1,3^, Qi Fan^1^, Xiaomei Zhang^1,3,4^, Xiaolong Cui^2*^ and Hong Yu^1*^


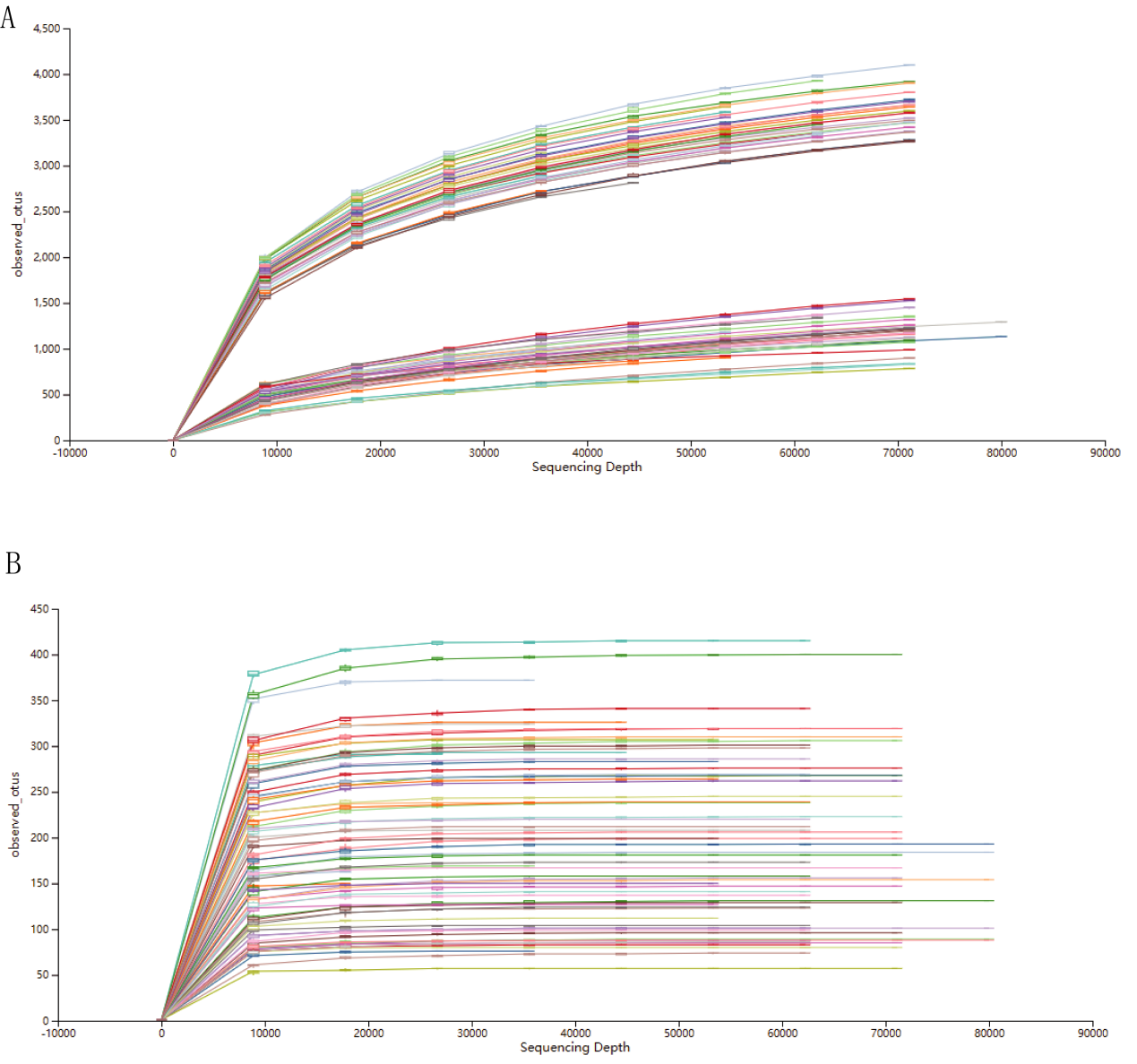


Additional file 2: Fig. S2. Rarefaction curves of the bacterial (A) and fungal (B) communities collected from fruiting bodies of *O. highlandensis* and the surrounding soil.
