# Supplementary material for: Endogenous bacteria inhabiting the Ophiocordyceps highlandensis during fruiting body development
Source: BMC Microbiol. 2021 Jun 11;21:178. doi: 10.1186/s12866-021-02227-w (PMC8196446; doi:10.1186/s12866-021-02227-w)
Supplement: Supplementary file 3 — Additional file 3: Fig. S3. Box plot of multivariate alpha dispersions of the microbial composition. (A) Comparisons of the bacterial alpha dispersions (Evenness parameter) grouped with multivariate variables. (B) Comparisons of the fungal alpha dispersions (Evenness parameter) grouped with multivariate variables. [file 12866_2021_2227_MOESM3_ESM.docx]

Endogenous bacteria inhabiting the *Ophiocordyceps highlandensis* during fruiting body development

Chengpeng Li^2#^, Dexiang Tang^1,2#^, Yuanbing Wang^1,3^, Qi Fan^1^, Xiaomei Zhang^1,3,4^, Xiaolong Cui^2*^ and Hong Yu^1*^


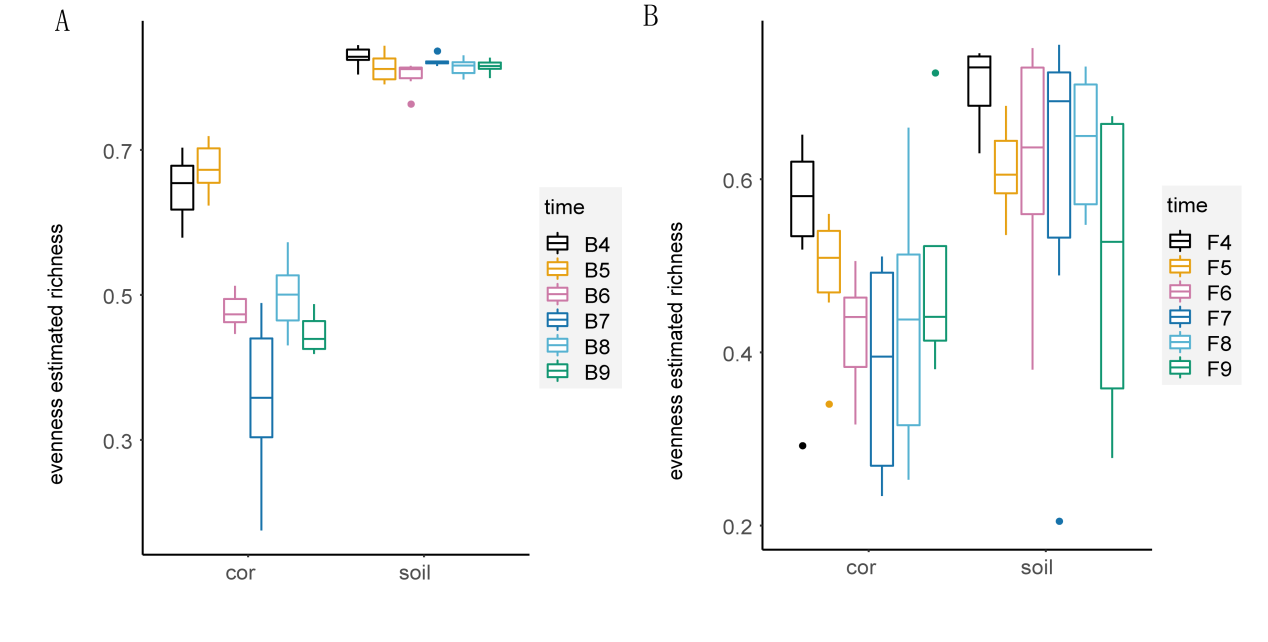


Additional file 3: Fig. S3. Box plot of multivariate alpha dispersions of the microbial composition. (A) Comparisons of the bacterial alpha dispersions (evenness parameter) grouped with multivariate variables. (B) Comparisons of the fungal alpha dispersions (evenness parameter) grouped with multivariate variables.
