# Supplementary material for: Endogenous bacteria inhabiting the Ophiocordyceps highlandensis during fruiting body development
Source: BMC Microbiol. 2021 Jun 11;21:178. doi: 10.1186/s12866-021-02227-w (PMC8196446; doi:10.1186/s12866-021-02227-w)
Supplement: Supplementary file 6 — Additional file 6: Fig. S6. Gel image for DNA extracted from soil; numbers 1–6 were collected in April and 14–19 in May. A and B were original gel image, the B was the second test result of sample 6 and 16, the sample and all procedures were same with the first test. C was processed in Power Point, numbers 1–5,14-15,17–19 of C were from A. numbers 6,16 of C were from B. Fig. S7. Gel image for DNA extracted from soil; numbers 1–6 were collected in June and 7–12 in July. A and B were original gel image, the B was the second test result of sample 4, the sample and all procedures were same with the first test. C was processed in Power Point, numbers 1–3,5–12 of C were from A, numbers 4 of C was from B. Fig. S8. Gel image for DNA extracted from soil; numbers 1–6 were collected in August. Fig. S9. Gel image for DNA extracted from soil; numbers 7–12 were collected in September. A was original gel image, B was processed in Power Point, numbers 7–12 of B were from A. [file 12866_2021_2227_MOESM6_ESM.docx]

Endogenous bacteria inhabiting the *Ophiocordyceps highlandensis* during fruiting body development

Chengpeng Li^2#^, Dexiang Tang^1,2#^, Yuanbing Wang^1,3^, Qi Fan^1^, Xiaomei Zhang^1,3,4^, Xiaolong Cui^2*^ and Hong Yu^1*^


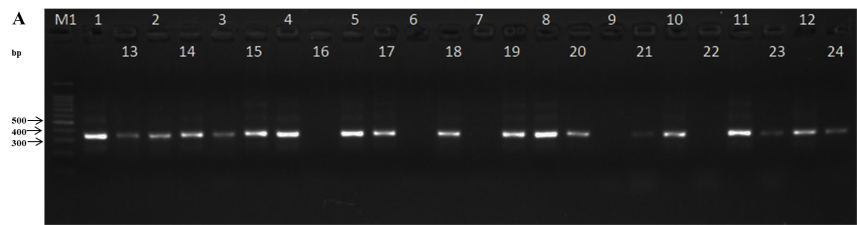

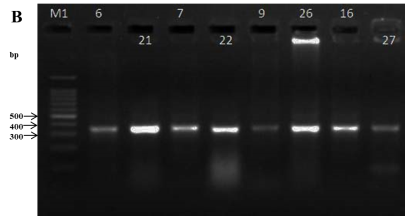


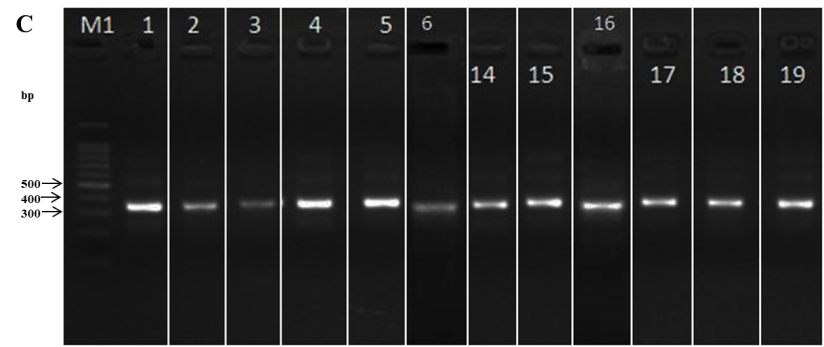


Additional file 6: Fig. S6. Gel image for DNA extracted from soil; numbers 1-6 were collected in April and 14-19 in May. A and B were [original](javascript:;) gel image, the B was the second test result of sample 6 and 16, the sample and all procedures were same with the first test. C was processed in Power Point, numbers 1-5,14-15,17-19 of C were from A. numbers 6,16 of C were from B.


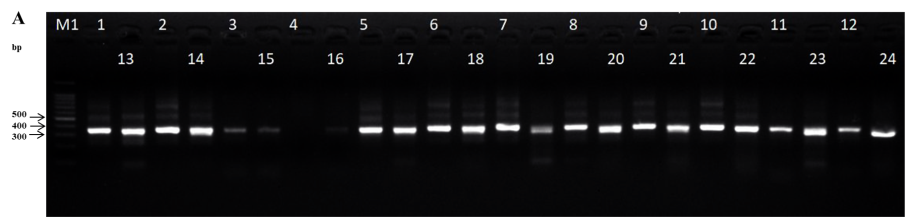

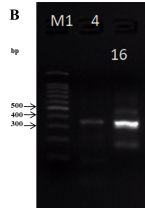


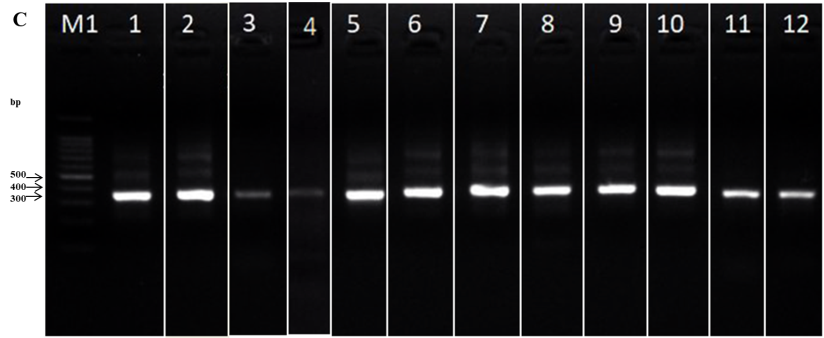


Additional file 6: Fig. S7. Gel image for DNA extracted from soil; numbers 1-6 were collected in June and 7-12 in July. A and B were [original](javascript:;) gel image, the B was the second test result of sample 4, the sample and all procedures were same with the first test. C was processed in Power Point, numbers 1-3,5-12 of C were from A, numbers 4 of C was from B.


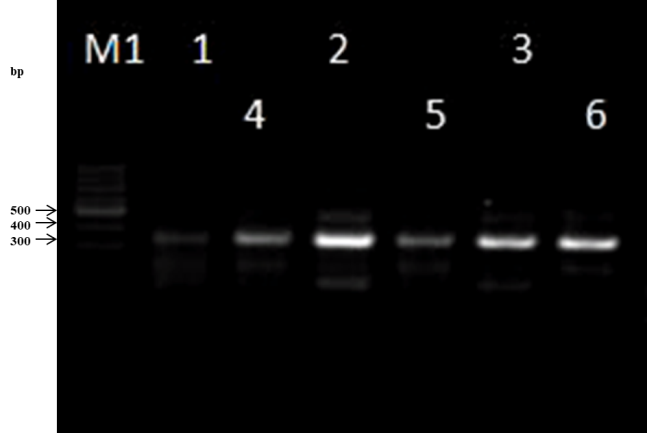


Additional file 6: Fig. S8. Gel image for DNA extracted from soil; numbers 1-6 were collected in August.


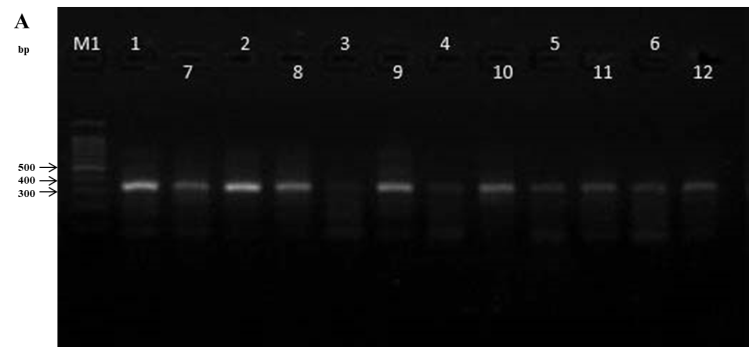

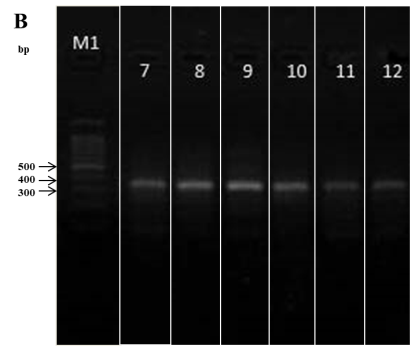


Additional file 6: Fig. S9. Gel image for DNA extracted from soil; numbers 7-12 were collected in September. A was [original](javascript:;) gel image, B was processed in Power Point, numbers 7-12 of B were from A.
