# Supplementary material for: Endogenous bacteria inhabiting the Ophiocordyceps highlandensis during fruiting body development
Source: BMC Microbiol. 2021 Jun 11;21:178. doi: 10.1186/s12866-021-02227-w (PMC8196446; doi:10.1186/s12866-021-02227-w)
Supplement: Supplementary file 7 — Additional file 7: Table S1. Summary of the sequences and richness and diversity indexes of the bacteria in the fruiting bodies. Table S2. Summary of the sequences and richness and diversity indexes of the bacteria in the soil microhabitat. Table S3. Summary of the sequences and richness and diversity indexes of the fungi in the fruiting bodies. Table S4. Summary of the sequences and richness and diversity indexes the fungi in the soil microhabitat. [file 12866_2021_2227_MOESM7_ESM.docx]

Endogenous bacteria inhabiting the *Ophiocordyceps highlandensis* during fruiting body development

Chengpeng Li^2#^, Dexiang Tang^1,2#^, Yuanbing Wang^1,3^, Qi Fan^1^, Xiaomei Zhang^1,3,4^, Xiaolong Cui^2*^ and Hong Yu^1*^

Additional file 7: Table S1. Summary of the sequences and richness and diversity indexes of the bacteria in the fruiting bodies.

| SampleID | Clean_reads | Group | Shannon | Evenness | SampleID | Clean_reads | Group | Shannon | Evenness |
| --- | --- | --- | --- | --- | --- | --- | --- | --- | --- |
| corB4r1 | 82647 | corB4 | 6.486 | 0.662 | corB7r1 | 80194 | corB7 | 3.972 | 0.321 |
| corB4r2 | 47490 | corB4 | 5.799 | 0.579 | corB7r2 | 80284 | corB7 | 3.925 | 0.395 |
| corB4r3 | 52455 | corB4 | 6.652 | 0.684 | corB7r3 | 80157 | corB7 | 4.830 | 0.489 |
| corB4r4 | 53394 | corB4 | 7.071 | 0.703 | corB7r4 | 80117 | corB7 | 4.744 | 0.455 |
| corB4r5 | 55145 | corB4 | 6.004 | 0.608 | corB7r5 | 80073 | corB7 | 4.222 | 0.298 |
| corB4r6 | 80144 | corB4 | 6.556 | 0.646 | corB7r6 | 80019 | corB7 | 4.361 | 0.175 |
| corB5r1 | 75990 | corB5 | 3.299 | 0.623 | corB8r1 | 80138 | corB8 | 6.134 | 0.509 |
| corB5r2 | 79396 | corB5 | 3.882 | 0.677 | corB8r2 | 71446 | corB8 | 6.636 | 0.491 |
| corB5r3 | 80170 | corB5 | 5.005 | 0.719 | corB8r3 | 69792 | corB8 | 7.030 | 0.573 |
| corB5r4 | 80057 | corB5 | 4.631 | 0.650 | corB8r4 | 93452 | corB8 | 6.471 | 0.456 |
| corB5r5 | 79699 | corB5 | 2.935 | 0.668 | corB8r5 | 81432 | corB8 | 6.627 | 0.430 |
| corB5r6 | 76435 | corB5 | 1.647 | 0.710 | corB8r6 | 76363 | corB8 | 7.003 | 0.533 |
| corB6r1 | 80021 | corB6 | 5.128 | 0.501 | corB9r1 | 80099 | corB9 | 4.965 | 0.422 |
| corB6r2 | 80116 | corB6 | 5.017 | 0.474 | corB9r2 | 80366 | corB9 | 4.688 | 0.419 |
| corB6r3 | 76720 | corB6 | 5.843 | 0.459 | corB9r3 | 61113 | corB9 | 4.569 | 0.487 |
| corB6r4 | 80158 | corB6 | 4.551 | 0.472 | corB9r4 | 63988 | corB9 | 4.651 | 0.471 |
| corB6r5 | 80128 | corB6 | 3.997 | 0.446 | corB9r5 | 56367 | corB9 | 4.428 | 0.437 |
| corB6r6 | 80188 | corB6 | 5.363 | 0.513 | corB9r6 | 61688 | corB9 | 5.002 | 0.442 |

Additional file 7: Table S2. Summary of the sequences and richness and diversity indexes of the bacteria in the soil microhabitat.

| SampleID | Clean_reads | Group | Shannon | Evenness | SampleID | Clean_reads | Group | Shannon | Evenness |
| --- | --- | --- | --- | --- | --- | --- | --- | --- | --- |
| soiB4r1 | 65097 | soiB4 | 9.926 | 0.845 | soiB7r1 | 80234 | soiB7 | 9.541 | 0.819 |
| soiB4r2 | 43627 | soiB4 | -- | -- | soiB7r2 | 80046 | soiB7 | 9.506 | 0.816 |
| soiB4r3 | 62181 | soiB4 | 9.833 | 0.838 | soiB7r3 | 80239 | soiB7 | 9.764 | 0.830 |
| soiB4r4 | 72669 | soiB4 | 9.288 | 0.804 | soiB7r4 | 80170 | soiB7 | 9.505 | 0.820 |
| soiB4r5 | 83003 | soiB4 | 9.666 | 0.829 | soiB7r5 | 71697 | soiB7 | 9.527 | 0.822 |
| soiB4r6 | 80187 | soiB4 | 9.724 | 0.824 | soiB7r6 | 79118 | soiB7 | 9.567 | 0.821 |
| soiB5r1 | 82226 | soiB5 | 9.133 | 0.796 | soiB8r1 | 61351 | soiB8 | 9.714 | 0.830 |
| soiB5r2 | 80124 | soiB5 | 9.265 | 0.802 | soiB8r2 | 80110 | soiB8 | 9.539 | 0.822 |
| soiB5r3 | 46046 | soiB5 | 9.082 | 0.791 | soiB8r3 | 80142 | soiB8 | 9.150 | 0.797 |
| soiB5r4 | 83930 | soiB5 | 9.922 | 0.844 | soiB8r4 | 80320 | soiB8 | 9.444 | 0.815 |
| soiB5r5 | 68344 | soiB5 | 9.536 | 0.821 | soiB8r5 | 80028 | soiB8 | 9.445 | 0.818 |
| soiB5r6 | 70846 | soiB5 | 9.763 | 0.828 | soiB8r6 | 80149 | soiB8 | 9.333 | 0.803 |
| soiB6r1 | 80296 | soiB6 | 9.423 | 0.811 | soiB9r1 | 54771 | soiB9 | 9.136 | 0.799 |
| soiB6r2 | 80122 | soiB6 | 9.548 | 0.816 | soiB9r2 | 69380 | soiB9 | 9.480 | 0.821 |
| soiB6r3 | 83136 | soiB6 | 9.481 | 0.811 | soiB9r3 | 80240 | soiB9 | 9.619 | 0.827 |
| soiB6r4 | 80285 | soiB6 | 9.162 | 0.795 | soiB9r4 | 62209 | soiB9 | 9.530 | 0.819 |
| soiB6r5 | 80223 | soiB6 | 8.683 | 0.757 | soiB9r5 | 47863 | soiB9 | 9.387 | 0.812 |
| soiB6r6 | 80140 | soiB6 | 9.395 | 0.814 | soiB9r6 | 56063 | soiB9 | 9.418 | 0.812 |

Additional file 7: Table S3. Summary of the sequences and richness and diversity indexes of the fungi in the fruiting bodies.

| SampleID | Clean_reads | Group | Shannon | Evenness | SampleID | Clean_reads | Group | Shannon | Evenness |
| --- | --- | --- | --- | --- | --- | --- | --- | --- | --- |
| corF4r1 | 54011 | corF4 | 4.726 | 0.581 | corF7r1 | 72161 | corF7 | 3.995 | 0.494 |
| corF4r2 | 41099 | corF4 | 4.431 | 0.519 | corF7r2 | 77305 | corF7 | 3.889 | 0.511 |
| corF4r3 | 47584 | corF4 | 5.289 | 0.634 | corF7r3 | 68553 | corF7 | 1.958 | 0.305 |
| corF4r4 | 55649 | corF4 | 5.139 | 0.651 | corF7r4 | 79247 | corF7 | 3.514 | 0.486 |
| corF4r5 | 83998 | corF4 | 1.994 | 0.287 | corF7r5 | 78578 | corF7 | 1.686 | 0.257 |
| corF4r6 | 43370 | corF4 | 4.291 | 0.580 | corF7r6 | 68724 | corF7 | 1.439 | 0.234 |
| corF5r1 | 67548 | corF5 | 3.679 | 0.504 | corF8r1 | 72333 | corF8 | 1.827 | 0.285 |
| corF5r2 | 82556 | corF5 | 2.165 | 0.335 | corF8r2 | 70031 | corF8 | 3.727 | 0.528 |
| corF5r3 | 68185 | corF5 | 3.280 | 0.514 | corF8r3 | 68425 | corF8 | 3.121 | 0.468 |
| corF5r4 | 80903 | corF5 | 3.519 | 0.549 | corF8r4 | 66923 | corF8 | 5.079 | 0.660 |
| corF5r5 | 70640 | corF5 | 3.028 | 0.458 | corF8r5 | 78307 | corF8 | 2.380 | 0.408 |
| corF5r6 | 83589 | corF5 | 3.719 | 0.560 | corF8r6 | 76928 | corF8 | 1.600 | 0.253 |
| corF6r1 | 76025 | corF6 | 2.585 | 0.371 | corF9r1 | 31479 | corF9 | 5.875 | 0.718 |
| corF6r2 | 63172 | corF6 | 3.203 | 0.464 | corF9r2 | 11497 | corF9 | -- | -- |
| corF6r3 | 77543 | corF6 | 3.306 | 0.462 | corF9r3 | 42138 | corF9 | 2.378 | 0.381 |
| corF6r4 | 66834 | corF6 | 2.762 | 0.419 | corF9r4 | 24876 | corF9 | 3.120 | 0.425 |
| corF6r5 | 70964 | corF6 | 3.485 | 0.505 | corF9r5 | 22985 | corF9 | 3.309 | 0.458 |
| corF6r6 | 69700 | corF6 | 2.036 | 0.317 | corF9r6 | 6716 | corF9 | -- | -- |

Additional file 7: Table S4. Summary of the sequences and richness and diversity indexes the fungi in the soil microhabitat.

| SampleID | Clean_reads | Group | Shannon | Evenness | SampleID | Clean_reads | Group | Shannon | Evenness |
| --- | --- | --- | --- | --- | --- | --- | --- | --- | --- |
| soiF4r1 | 74255 | soiF4 | 5.407 | 0.672 | soiF7r1 | 62236 | soiF7 | 5.325 | 0.663 |
| soiF4r2 | 75105 | soiF4 | 5.722 | 0.725 | soiF7r2 | 85460 | soiF7 | 1.447 | 0.200 |
| soiF4r3 | 70978 | soiF4 | 6.476 | 0.746 | soiF7r3 | 72854 | soiF7 | 6.510 | 0.755 |
| soiF4r4 | 75377 | soiF4 | 5.796 | 0.745 | soiF7r4 | 69711 | soiF7 | 3.849 | 0.489 |
| soiF4r5 | 75454 | soiF4 | 5.913 | 0.734 | soiF7r5 | 73911 | soiF7 | 6.017 | 0.725 |
| soiF4r6 | 68611 | soiF4 | 5.068 | 0.630 | soiF7r6 | 72523 | soiF7 | 5.955 | 0.718 |
| soiF5r1 | 82232 | soiF5 | 4.625 | 0.611 | soiF8r1 | 61701 | soiF8 | 3.952 | 0.547 |
| soiF5r2 | 81980 | soiF5 | 4.334 | 0.579 | soiF8r2 | 67207 | soiF8 | 5.289 | 0.680 |
| soiF5r3 | 66169 | soiF5 | 4.724 | 0.600 | soiF8r3 | 60741 | soiF8 | 5.565 | 0.730 |
| soiF5r4 | 73765 | soiF5 | 5.653 | 0.685 | soiF8r4 | 67783 | soiF8 | 5.543 | 0.719 |
| soiF5r5 | 71859 | soiF5 | 4.013 | 0.536 | soiF8r5 | 60662 | soiF8 | 4.330 | 0.620 |
| soiF5r6 | 79860 | soiF5 | 5.382 | 0.656 | soiF8r6 | 71436 | soiF8 | 4.092 | 0.555 |
| soiF6r1 | 67423 | soiF6 | 6.304 | 0.752 | soiF9r1 | 68336 | soiF9 | 2.992 | 0.403 |
| soiF6r2 | 73459 | soiF6 | 2.913 | 0.380 | soiF9r2 | 36521 | soiF9 | 5.569 | 0.668 |
| soiF6r3 | 72152 | soiF6 | 4.424 | 0.554 | soiF9r3 | 54594 | soiF9 | 5.388 | 0.653 |
| soiF6r4 | 67593 | soiF6 | 4.700 | 0.577 | soiF9r4 | 61234 | soiF9 | 2.334 | 0.343 |
| soiF6r5 | 64970 | soiF6 | 6.078 | 0.740 | soiF9r5 | 49910 | soiF9 | 5.510 | 0.673 |
| soiF6r6 | 70040 | soiF6 | 5.709 | 0.696 | soiF9r6 | 65087 | soiF9 | 1.979 | 0.278 |
