# Supplementary material for: Endogenous bacteria inhabiting the Ophiocordyceps highlandensis during fruiting body development
Source: BMC Microbiol. 2021 Jun 11;21:178. doi: 10.1186/s12866-021-02227-w (PMC8196446; doi:10.1186/s12866-021-02227-w)
Supplement: Supplementary file 8 — Additional file 8: Table S5. Pairwise comparisons of the Shannon index and Evenness parameter among the bacterial groups inhabiting the soil microhabitat. The bold values represent statistically significant results. Table S6. Pairwise comparisons of the Shannon index and evenness parameter among the fungal groups inhabiting the fruiting body. The bold values indicate statistically significant results. Table S7. Pairwise comparisons of the Shannon index and Evenness parameter among the fungal groups inhabiting the soil microhabitat. The bold values represent statistically significant results. [file 12866_2021_2227_MOESM8_ESM.docx]

Endogenous bacteria inhabiting the *Ophiocordyceps highlandensis* during fruiting body development

Chengpeng Li^2#^, Dexiang Tang^1,2#^, Yuanbing Wang^1,3^, Qi Fan^1^, Xiaomei Zhang^1,3,4^, Xiaolong Cui^2*^ and Hong Yu^1*^

Additional file 8: Table S5. Pairwise comparisons of the Shannon index and evenness parameter among the bacterial groups inhabiting the soil microhabitat. The bold values represent statistically significant results.

| Shannon  Evenness  Evenness | soiB4 | soiB5 | soiB6 | soiB7 | soiB8 | soiB9 |
| --- | --- | --- | --- | --- | --- | --- |
| soiB4 |  | 0.233 | 0.063 | 0.233 | 0.132 | 0.091 |
| soiB5 | 0.176 |  | 0.661 | 0.565 | 0.886 | 1.000 |
| soiB6 | 0.061 | 0.458 |  | 0.036 | 0.565 | 0.661 |
| soiB7 | 0.229 | 0.661 | 0.010 |  | 0.134 | 0.134 |
| soiB8 | 0.127 | 0.772 | 0.179 | 0.229 |  | 0.886 |
| soiB9 | 0.127 | 1.000 | 0.136 | 0.289 | 1.000 |  |

Additional file 8: Table S6. Pairwise comparisons of the Shannon index and evenness parameter among the fungal groups inhabiting the fruiting body. The bold values indicate statistically significant results.

| Shannon  Evenness | corF4 | corF5 | corF6 | corF7 | corF8 | corF9 |
| --- | --- | --- | --- | --- | --- | --- |
| corF4 |  | 0.273 | 0.273 | 0.245 | 0.293 | 0.860 |
| corF5 | 0.410 |  | 0.601 | 0.860 | 0.860 | 1.000 |
| corF6 | 0.391 | 0.449 |  | 1.000 | 0.936 | 0.860 |
| corF7 | 0.375 | 0.391 | 0.802 |  | 1.000 | 0.860 |
| corF8 | 0.500 | 0.773 | 0.873 | 0.773 |  | 0.860 |
| corF9 | 0.773 | 0.773 | 0.773 | 0.773 | 0.773 |  |

Additional file 8: Table S7. Pairwise comparisons of the Shannon index and evenness parameter among the fungal groups inhabiting the soil microhabitat. The bold values represent statistically significant results.

| Shannon  Evenness | soiF4 | soiF5 | soiF6 | soiF7 | soiF8 | soiF9 |
| --- | --- | --- | --- | --- | --- | --- |
| soiF4 |  | 0.187 | 0.712 | 0.935 | 0.187 | 0.187 |
| soiF5 | 0.187 |  | 0.712 | 0.712 | 0.864 | 0.789 |
| soiF6 | 0.631 | 0.936 |  | 1.000 | 0.712 | 0.712 |
| soiF7 | 0.631 | 0.635 | 1.000 |  | 0.712 | 0.712 |
| soiF8 | 0.391 | 0.635 | 1.000 | 1.000 |  | 0.712 |
| soiF9 | 0.187 | 0.712 | 0.601 | 0.631 | 0.561 |  |
