# Supplementary material for: Endogenous bacteria inhabiting the Ophiocordyceps highlandensis during fruiting body development
Source: BMC Microbiol. 2021 Jun 11;21:178. doi: 10.1186/s12866-021-02227-w (PMC8196446; doi:10.1186/s12866-021-02227-w)
Supplement: Supplementary file 9 — Additional file 9: Table S8. Results of a pairwise permutational MANOVA on the beta diversity of the bacterial community composition in the fruiting body. ​P-values result from 999 permutations of a Bray-Curtis dissimilarity matrix. The bold values indicate statistically significant results. [file 12866_2021_2227_MOESM9_ESM.docx]

Endogenous bacteria inhabiting the *Ophiocordyceps highlandensis* during fruiting body development

Chengpeng Li^2#^, Dexiang Tang^1,2#^, Yuanbing Wang^1,3^, Qi Fan^1^, Xiaomei Zhang^1,3,4^, Xiaolong Cui^2*^ and Hong Yu^1*^

Additional file 9: Table S8. Results of a pairwise permutational MANOVA on the beta diversity of the bacterial community composition in the fruiting body. P-values result from 999 permutations of a Bray-Curtis dissimilarity matrix. The bold values indicate statistically significant results.

| Beta diversity  Beta diversity | corB4 | corB5 | corB6 | corB7 | corB8 | corB9 |
| --- | --- | --- | --- | --- | --- | --- |
| corB4 |  | 0.005 | 0.005 | 0.005 | 0.005 | 0.005 |
| corB5 | 0.005 |  | 0.005 | 0.005 | 0.005 | 0.005 |
| corB6 | 0.005 | 0.005 |  | 0.027 | 0.005 | 0.005 |
| corB7 | 0.005 | 0.005 | 0.027 |  | 0.005 | 0.005 |
| corB8 | 0.005 | 0.005 | 0.005 | 0.005 |  | 0.005 |
| corB9 | 0.005 | 0.005 | 0.005 | 0.005 | 0.005 |  |
