# Supplementary material for: Endogenous bacteria inhabiting the Ophiocordyceps highlandensis during fruiting body development
Source: BMC Microbiol. 2021 Jun 11;21:178. doi: 10.1186/s12866-021-02227-w (PMC8196446; doi:10.1186/s12866-021-02227-w)
Supplement: Supplementary file 10 — Additional file 10: Table S9. Relative abundances of the bacterial phyla and classes belonging to the Proteobacteria in all the fruiting body samples. Table S10. The relative abundances of the bacterial phyla and classes belonging to the Proteobacteria in all the soil microhabitat samples. Table S11. The relative abundances of the fungal phyla in all the fruiting body samples. Table S12. The relative abundances of the fungal phyla in all the soil microhabitat samples. [file 12866_2021_2227_MOESM10_ESM.docx]

Endogenous bacteria inhabiting the *Ophiocordyceps highlandensis* during fruiting body development

Chengpeng Li^2#^, Dexiang Tang^1,2#^, Yuanbing Wang^1,3^, Qi Fan^1^, Xiaomei Zhang^1,3,4^, Xiaolong Cui^2*^ and Hong Yu^1*^

Additional file 10: Table S9. Relative abundances of the bacterial phyla and classes belonging to the Proteobacteria in all the fruiting body samples.

| Samples | Alphaproteobacteria | Gammaproteobacteria | Firmicutes | Bacteroidetes | Actinobacteria | Deltaproteobacteria | Verrucomicrobia | Tenericutes | Synergistetes | Low Abundance |
| --- | --- | --- | --- | --- | --- | --- | --- | --- | --- | --- |
| corB4 | 0.571 | 3.328 | 70.711 | 13.701 | 1.875 | 5.211 | 0.136 | 2.503 | 1.005 | 0.959 |
| corB5 | 2.733 | 9.828 | 47.554 | 23.899 | 3.008 | 6.489 | 2.954 | 0.922 | 1.490 | 1.123 |
| corB6 | 29.722 | 29.391 | 5.516 | 22.981 | 5.397 | 2.621 | 2.320 | 0.419 | 0.147 | 1.485 |
| corB7 | 57.161 | 24.079 | 2.130 | 8.708 | 3.600 | 1.632 | 0.897 | 0.093 | 0.023 | 1.675 |
| corB8 | 25.448 | 27.020 | 1.213 | 40.680 | 3.656 | 0.338 | 0.365 | 0.140 | 0.000 | 1.139 |
| corB9 | 33.469 | 41.278 | 6.755 | 16.809 | 0.667 | 0.191 | 0.127 | 0.208 | 0.002 | 0.493 |

Additional file 10: Table S10. The relative abundances of the bacterial phyla and classes belonging to the Proteobacteria in all the soil microhabitat samples.

| Samples | Alphaproteobacteria | Acidobacteria | Gammaproteobacteria | Actinobacteria | Chloroflexi | Deltaproteobacteria | Planctomycetes | Bacteroidetes | Gemmatimonadetes | Low Abundance |
| --- | --- | --- | --- | --- | --- | --- | --- | --- | --- | --- |
| soiB4 | 18.422 | 16.216 | 12.482 | 11.761 | 7.309 | 7.201 | 6.245 | 5.590 | 4.515 | 10.260 |
| soiB5 | 13.146 | 19.715 | 11.628 | 11.588 | 11.475 | 7.195 | 6.252 | 3.241 | 4.372 | 11.387 |
| soiB6 | 25.017 | 19.301 | 11.074 | 9.786 | 6.942 | 6.383 | 5.963 | 4.615 | 3.621 | 7.298 |
| soiB7 | 23.804 | 19.031 | 11.123 | 11.174 | 6.476 | 6.671 | 4.496 | 4.952 | 4.088 | 8.186 |
| soiB8 | 19.182 | 18.430 | 10.605 | 12.620 | 9.172 | 7.939 | 3.763 | 4.657 | 4.794 | 8.839 |
| soiB9 | 25.869 | 16.594 | 12.558 | 10.795 | 5.996 | 6.654 | 3.799 | 5.898 | 3.766 | 8.071 |

Additional file 10: Table S11. The relative abundances of the fungal phyla in all the fruiting body samples.

| Samples | Ascomycota | Basidiomycota | Mortierellomycota | Mucoromycota | Chytridiomycota | Rozellomycota | unidentified | Glomeromycota | Blastocladiomycota | Low Abundance |
| --- | --- | --- | --- | --- | --- | --- | --- | --- | --- | --- |
| corF4 | 78.927 | 18.927 | 1.016 | 0.468 | 0.515 | 0.066 | 0.074 | 0.000 | 0.004 | 0.004 |
| corF5 | 70.268 | 8.178 | 8.268 | 13.285 | 0.000 | 0.000 | 0.000 | 0.000 | 0.000 | 0.000 |
| corF6 | 84.062 | 1.847 | 12.489 | 1.574 | 0.009 | 0.000 | 0.020 | 0.000 | 0.000 | 0.000 |
| corF7 | 90.092 | 8.178 | 1.063 | 0.655 | 0.004 | 0.009 | 0.000 | 0.000 | 0.000 | 0.000 |
| corF8 | 78.147 | 21.555 | 0.170 | 0.112 | 0.004 | 0.000 | 0.000 | 0.012 | 0.000 | 0.000 |
| corF9 | 82.218 | 10.436 | 3.988 | 0.732 | 1.661 | 0.651 | 0.309 | 0.000 | 0.004 | 0.000 |

Additional file 10: Table S12. The relative abundance of fungal phyla in all microhabitat soil samples.

| Samples | Ascomycota | Basidiomycota | Mortierellomycota | Blastocladiomycota | Chytridiomycota | unidentified | Rozellomycota | Mucoromycota | Calcarisporiellomycota | Low Abundance |
| --- | --- | --- | --- | --- | --- | --- | --- | --- | --- | --- |
| soiF4 | 62.753 | 32.076 | 4.423 | 0.000 | 0.217 | 0.010 | 0.324 | 0.118 | 0.071 | 0.008 |
| soiF5 | 49.551 | 46.963 | 2.769 | 0.052 | 0.230 | 0.006 | 0.184 | 0.173 | 0.031 | 0.040 |
| soiF6 | 38.511 | 48.678 | 12.336 | 0.000 | 0.292 | 0.023 | 0.031 | 0.125 | 0.002 | 0.002 |
| soiF7 | 42.199 | 43.871 | 12.807 | 0.001 | 0.256 | 0.788 | 0.027 | 0.046 | 0.000 | 0.005 |
| soiF8 | 43.004 | 54.493 | 2.186 | 0.015 | 0.033 | 0.076 | 0.114 | 0.033 | 0.018 | 0.028 |
| soiF9 | 45.478 | 50.717 | 1.895 | 1.324 | 0.215 | 0.159 | 0.171 | 0.000 | 0.000 | 0.040 |
