# Supplementary material for: Endogenous bacteria inhabiting the Ophiocordyceps highlandensis during fruiting body development
Source: BMC Microbiol. 2021 Jun 11;21:178. doi: 10.1186/s12866-021-02227-w (PMC8196446; doi:10.1186/s12866-021-02227-w)
Supplement: Supplementary file 11 — Additional file 11: Table S13. Distribution of the distinct bacterial functions during O. highlandensis maturation. [file 12866_2021_2227_MOESM11_ESM.docx]

Endogenous bacteria inhabiting the *Ophiocordyceps highlandensis* during fruiting body development

Chengpeng Li^2#^, Dexiang Tang^1,2#^, Yuanbing Wang^1,3^, Qi Fan^1^, Xiaomei Zhang^1,3,4^, Xiaolong Cui^2*^ and Hong Yu^1*^

Additional file 11: Table S13. Distribution of the distinct bacterial functions during *O. highlandensis* maturation.

| Category | Log value | Groups | LDA | Pathway | Description | Num |
| --- | --- | --- | --- | --- | --- | --- |
| PWY_5100 | 3.935 | corB4 | 3.593 | PWY-5100 | pyruvate fermentation to acetate and lactate II | 193.000 |
| PWY_5104 | 3.999 | corB4 | 3.496 | PWY-5104 | L-isoleucine biosynthesis IV | 190.000 |
| P161_PWY | 3.824 | corB4 | 3.479 | P161-PWY | acetylene degradation | 16.000 |
| GLYCOCAT_PWY | 3.917 | corB4 | 3.469 | GLYCOCAT-PWY | glycogen degradation I (bacterial) | 247.000 |
| GLYCOGENSYNTH_PWY | 3.914 | corB4 | 3.441 | GLYCOGENSYNTH-PWY | glycogen biosynthesis I (from ADP-D-Glucose) | 93.000 |
| P42_PWY | 3.833 | corB5 | 3.318 | P42-PWY | incomplete reductive TCA cycle | 140.000 |
| PWY_6895 | 3.557 | corB5 | 3.247 | PWY-6895 | superpathway of thiamin diphosphate biosynthesis II | 173.000 |
| DENOVOPURINE2_PWY | 3.794 | corB5 | 3.237 | DENOVOPURINE2-PWY | superpathway of purine nucleotides de novo biosynthesis II | 183.000 |
| PWY_6545 | 3.511 | corB5 | 3.192 | PWY-6545 | pyrimidine deoxyribonucleotides de novo biosynthesis III | 170.000 |
| PWY_7187 | 3.689 | corB5 | 3.134 | PWY-7187 | pyrimidine deoxyribonucleotides de novo biosynthesis II | 2.000 |
| P23_PWY | 3.763 | corB6 | 3.351 | P23-PWY | reductive TCA cycle I | 174.000 |
| PWY_7211 | 3.723 | corB6 | 3.242 | PWY-7211 | superpathway of pyrimidine deoxyribonucleotides de novo biosynthesis | 265.000 |
| PYRIDOXSYN_PWY | 3.587 | corB6 | 3.171 | PYRIDOXSYN-PWY | pyridoxal 5'-phosphate biosynthesis I | 109.000 |
| NAGLIPASYN_PWY | 3.672 | corB6 | 3.145 | NAGLIPASYN-PWY | superpathway of (Kdo)2-lipid A biosynthesis | 273.000 |
| PWY_6467 | 3.665 | corB6 | 3.129 | PWY-6467 | Kdo transfer to lipid IVA III (Chlamydia) | 17.000 |
| P105_PWY | 3.774 | corB7 | 3.361 | P105-PWY | TCA cycle IV (2-oxoglutarate decarboxylase) | 153.000 |
| TCA_GLYOX_BYPASS | 3.659 | corB7 | 3.300 | TCA-GLYOX-BYPASS | superpathway of glycolysis, pyruvate dehydrogenase, TCA, and glyoxylate bypass | 168.000 |
| BIOTIN_BIOSYNTHESIS_PWY | 3.648 | corB7 | 3.292 | BIOTIN-BIOSYNTHESIS-PWY | biotin biosynthesis I | 67.000 |
| REDCITCYC | 3.814 | corB7 | 3.282 | REDCITCYC | TCA cycle VIII (helicobacter) | 45.000 |
| GLYCOLYSIS_TCA_GLYOX_BYPASS | 3.680 | corB7 | 3.278 | GLYCOLYSIS-TCA-GLYOX-BYPASS | superpathway of glycolysis, pyruvate dehydrogenase, TCA, and glyoxylate bypass | 113.000 |
| PWY_3781 | 4.185 | corB8 | 3.798 | PWY-3781 | aerobic respiration I (cytochrome c) | 276.000 |
| PWY_5345 | 3.787 | corB8 | 3.374 | PWY-5345 | superpathway of L-methionine biosynthesis (by sulfhydrylation) | 188.000 |
| HSERMETANA_PWY | 3.788 | corB8 | 3.346 | HSERMETANA-PWY | L-methionine biosynthesis III | 15.000 |
| SO4ASSIM_PWY | 3.716 | corB8 | 3.330 | SO4ASSIM-PWY | sulfate reduction I (assimilatory) | 294.000 |
| PWY0_1586 | 3.880 | corB8 | 3.312 | PWY0-1586 | peptidoglycan maturation (meso-diaminopimelate containing) | 179.000 |
| PWYG_321 | 3.941 | corB9 | 3.613 | PWYG-321 | mycolate biosynthesis | 237.000 |
| PWY_5989 | 3.928 | corB9 | 3.602 | PWY-5989 | stearate biosynthesis II (bacteria and plants) | 241.000 |
| PWY_7664 | 3.922 | corB9 | 3.594 | PWY-7664 | oleate biosynthesis IV (anaerobic) | 196.000 |
| PWY_6282 | 3.915 | corB9 | 3.590 | PWY-6282 | palmitoleate biosynthesis I (from (5Z)-dodec-5-enoate) | 110.000 |
| PWY_7094 | 3.927 | corB9 | 3.569 | PWY-7094 | fatty acid salvage | 5.000 |
