# Supplementary material for: Endogenous bacteria inhabiting the Ophiocordyceps highlandensis during fruiting body development
Source: BMC Microbiol. 2021 Jun 11;21:178. doi: 10.1186/s12866-021-02227-w (PMC8196446; doi:10.1186/s12866-021-02227-w)
Supplement: Supplementary file 12 — Additional file 12: Table S14. Taxonomic summary of 4 supercore microorganisms. [file 12866_2021_2227_MOESM12_ESM.docx]

Endogenous bacteria inhabiting the *Ophiocordyceps highlandensis* during fruiting body development

Chengpeng Li^2#^, Dexiang Tang^1,2#^, Yuanbing Wang^1,3^, Qi Fan^1^, Xiaomei Zhang^1,3,4^, Xiaolong Cui^2*^ and Hong Yu^1*^

Additional file 12: Table S14 Taxonomic summary of 4 supercore microorganisms.

| OUT_ID | Kingdom | Phylum | Class | Order | Family | Genus | species | Source |
| --- | --- | --- | --- | --- | --- | --- | --- | --- |
| OTU_5 | Bacteria | Proteobacteria | Gammaproteobacteria | Pseudomonadales | Pseudomonadaceae | *Pseudomonas* | *Pseudomonas* sp. (strain C2) | Stem of *Ziziphus jujuba Mill* |
| OTU_9 | Bacteria | Proteobacteria | Alphaproteobacteria | Rhizobiales | Xanthobacteraceae | *Bradyrhizobium* | *Bradyrhizobium* sp. (strain LCT2) | Root nodule of *Lespedeza cuneata* |
| OTU_105 | Bacteria | Proteobacteria | Alphaproteobacteria | Rhizobiales | Xanthobacteraceae | uncultured | __ | __ |
| OTU_144 | Bacteria | Actinobacteria | Actinobacteria | Corynebacteriales | Mycobacteriaceae | *Mycobacterium* | *Mycolicibacterium madagascariense*（JCM 13574） | *Sphagnum obtusiusculum* |
